# Strontium and oxygen isotope analysis reveals changing connections to place and group membership in the world’s earliest village societies

**DOI:** 10.1038/s41598-025-18134-3

**Published:** 2025-10-03

**Authors:** Jo-Hannah Plug, Kelly E. Blevins, Frédéric Abbès, Peter M. M. G. Akkermans, Anna M. Bach Gómez, Marie-Laure Chambrade, Bérénice Chamel, Eric Coqueugniot, Miquel Molist, Marie Orange, Johannes van der Plicht, Stamatia Galata, Geoffrey Nowell, Janet Montgomery, Jessica Pearson, Eva Fernández-Domínguez

**Affiliations:** 1 Department of Archaeology, Classics and Egyptology, University of Liverpool, Liverpool, UK; 2 Department of Archaeology, Durham University, Durham, UK; 3 Center for Bioarchaeological Research, Arizona State University, Tempe, USA; 4 Maison de l’Orient et de la Méditerranée CNRS - Université Lyon 2, Lyon, France; 5 Faculty of Archaeology, Leiden University, Leiden, The Netherlands; 6 Department de Prehistòria, Universitat Autònoma de Barcelona, Bellaterra, Spain; 7 CEPAM – UMR 7264, CNRS, Nice, France; 8 Institut Français du Proche-Orient, Beirut, Lebanon; 9 Department of Archaeology, Classics and History, University of New England, Armidale, Australia; 10 Faculty of Science and Engineering, University of Groningen, Groningen, The Netherlands; 11 School of Biological and Environmental Sciences, Liverpool John Moores University, Liverpool, UK

**Keywords:** Neolithic, Upper Mesopotamia, Syria, Mobility, Sr & O Isotope Analysis, Group membership, Psychology and behaviour, Socioeconomic scenarios, Population dynamics, Stable isotope analysis

## Abstract

The Neolithic of southwest Asia, 11,600–7500 years ago, charts the earliest establishment of permanent settlements and changes in food procurement and community structure that transformed human lifeways. Our understanding of the social behaviors that impacted these shifting connections to place and group membership can be improved by studying how people moved across landscapes. Parts of southwest Asia have shown contrasting evidence for mobility practices, but little is known from the Northern Levant, a region key to the development and transmission of agriculture and settled life, particularly for the latest Neolithic stages. We measured strontium and oxygen isotope values in 71 human teeth from five archeological sites in Syria, spanning the entire Neolithic period. A shift to broadly local communities following the establishment of village life suggests consolidation of group membership and deep connections to particular locales, perhaps aimed at social cohesion. Mobility then increases in the later Neolithic, explaining the high degree of cross-regional connectivity witnessed archeologically. A sex-bias towards female mobility during this period may point towards the formation of patrilocal traditions. At our sites both non-local and local individuals were afforded similar burial treatment, suggesting inclusivity in group membership and mobile individuals connecting to new places in the landscape.

**Supplementary Information:**

The online version contains supplementary material available at 10.1038/s41598-025-18134-3.

## Introduction

The Neolithic of southwest Asia is marked by pivotal changes in human behavior, most notably the transition from mobile hunter-gatherer to sedentary agricultural lifestyles^1–4^. In northern Syria, the earliest Neolithic, the Pre-Pottery Neolithic A (PPNA) in the 10th–9th millennia BCE, is associated with investment in semi-permanent structures, establishment of small, increasingly sedentary communities and initial management of various wild plant and animal species^5,6^. The subsequent Pre-Pottery Neolithic B (PPNB) in the 9th–7th millennia BCE is characterized by the gradual domestication of animals and plants, the expansion of permanent settlements, an increase in population density, sedentarization of previously mobile groups and/or demographic growth^7^, the emergence of nomadic pastoralists^8^, and a florescence of ritual behavior^9,10^. The Late Neolithic (LN) in the 7th–6th millennia BCE is defined by the gradual establishment of pottery vessels as an important element of material culture, suitable for a range of storage and food preparation tasks, but also for the communication of group identities^4,11^. After the demographic growth and settlement stability of the PPNB, many sites were abandoned, leading to population dispersal into new areas and the formation of smaller villages with flexible economic systems that combined agriculture and pastoralism^12^.

In the Northern Levant, as in other parts of southwest Asia, connections to specific geographical locations evolved during the Neolithic. This unprecedented commitment to place is argued to have led to novel concepts of ownership and group identities^13–15^. At many early Neolithic sites there was a strong emphasis on house decoration and continuity of rebuilding houses on the same footprint over generations^15–17^. In the PPNB, communities participated in elaborate mortuary treatments, with deposition of the dead within houses reinforcing the importance of place and community cohesion^10,15,18^. This paradigm, however, changed towards the end of the Neolithic when the ritual investment in buildings decreased^2,19^ and although mortuary practices continued to be emphasized spatially through the clustering of graves or the use of collective mortuary features, certain burial customs such as the use of communal cemetery areas and cremation gained in popularity^20^. Recent bioarcheological studies show that mortuary treatment and the spatial organization of the dead may have been structured by factors such as biological or fictive kinship (e.g. task- or age-based groups) that may have differed according to life stage, location and region^21–23^, suggesting that group membership throughout the Neolithic of southwest Asia was complex.

Understanding how these aspects of human behavior changed across the Neolithic is critical since, in apparent contrast to what comes before and after, the PPNB is considered to be a period when many social and ritual practices promoting social cohesion and connection to place reach their zenith^9,10^. Despite evidence that communities of the Northern Levant and beyond were connected to one another through exchange networks from the earliest phases of the Neolithic onward^24–26^, it is still unclear how common the movement of people was into new areas, how permeable social groups were for new members, and in what ways these aspects of mobility intersected with wider Neolithic developments.

Strontium (Sr) and oxygen (O) isotope analysis of human tooth enamel has the unique potential to provide evidence of in-life mobility of individuals, which can be compared with others within and between sites to establish the nature of residential and logistical mobility. The ratios of these isotopes reflect different, complementary aspects of the locality in which the tissue was formed. Briefly, ^87^Sr/^86^Sr ratios in human tissues record the local geology predominantly where consumed foodstuffs grew, whereas δ^18^O values reflect waters ingested^27,28^. Bioavailable ^87^Sr/^86^Sr ratios vary between regions based on the age and composition of the local bedrock, as well as differential weathering and variable contributions of aeolian and fluvial sediments^28^. Environmental factors impacting the δ^18^O values of local waters include air temperature, precipitation, latitude, altitude, continental effects, evaporation, and water mixing^29^. Due to its resilience to diagenetic alteration and lack of remodeling, tooth enamel reflects accurately the locally bioavailable Sr and O at the time of tissue formation during childhood^28^. Individuals yielding ^87^Sr/^86^Sr ratios and/or δ^18^O values diverging from locally expected baselines established for their burial location can be interpreted as having spent their childhood elsewhere. However, a range of cultural factors such as boiling, brewing and stewing, as well as milk consumption, are also known to impact δ^18^O values^30,31^.

**Fig. 1:**
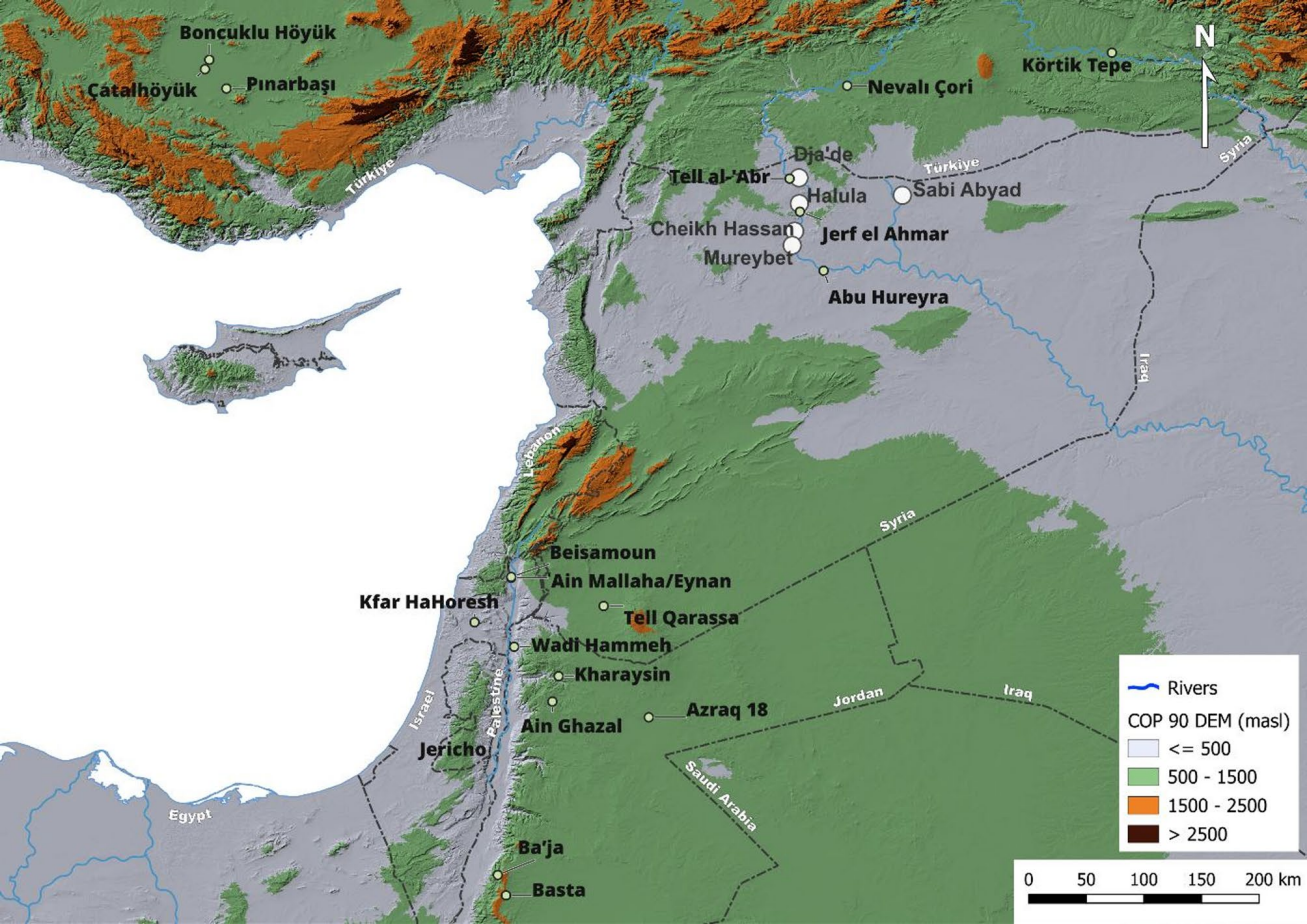
All sites mentioned in this paper. Large white circles indicate the sites analyzed in this study. Produced using Copernicus WorldDEM-30 © DLR e.V. 2010–2014 and © Airbus Defence and Space GmbH 2014–2018 provided under COPERNICUS by the European Union and ESA; all rights reserved.

Particularly over the past decade, Sr and O isotope analyses have been used to study mobility patterns at various Neolithic sites in southwest Asia^23,32–38^. In central Anatolia the data points towards little residential mobility through the 14th–9th millennia BCE followed by a subtle increase after the initial establishment of settled agropastoral lifestyles in the 7th millennium BCE during the rise of mega-site Çatalhöyük^23^. In contrast, an overall decrease in mobility during the earlier Neolithic, possibly reflecting the transition toward sedentism, has been observed in the Southern Levant and southeastern Anatolia^35,37,38^. There is a conspicuous absence, however, of empirical data from the Northern Levant and Syria especially—a key region for the origins of agriculture and settled life, connecting the Southern Levant and Anatolia. This limits our understanding of whether the patterns seen elsewhere form a continuum of practices across the southwest Asian Neolithic or point to more localized responses and decision-making in residential mobility. Important outstanding questions include: How mobile were people during the Neolithic in the Northern Levant? Does mobility change over the course of the Neolithic along with social, cultural and economic developments? To what extent did mobile individuals become integrated into the local communities (as evidenced through location and manner of burial)? Were specific groups (e.g. based on task, status or gendered differences) more likely to engage in mobile activities? To address these questions, we measured the Sr and O isotope ratios in tooth enamel of 71 individuals from five sites dating between the 9th and 6th millennia BCE (Late PPNA – LN) in northern Syria (Fig. 1, Table 1, Supplementary Table S1): Tell Cheikh Hassan (Cheikh Hassan), Tell Mureybet (Mureybet), Tell Dja’de el-Mughara (Dja’de), and Tell Halula (Halula) from the Euphrates and Tell Sabi Abyad (Sabi Abyad) from the Balikh, a tributary of the Euphrates. For site descriptions see Supplementary Notes.

**Table 1:** Sites, and period and calibrated BCE dates of samples reported here (see also Supplementary Note).

Site	Period	Dates (calibrated)
Cheikh Hassan	Late PPNA	~ 8900 BCE
Dja’de	Early PPNB	~ 8800–8290 BCE
Mureybet	Middle PPNB	~ 8300–8200 BCE
Halula	Middle-Late PPNB	~ 7550–7300 BCE
Sabi Abyad	Late Neolithic	~ 6400–5800 BCE

These sites were chosen to cover a time transect spanning the PPNA-LN cultural periods in the Northern Levant, thereby enabling us to connect variability in mobility behavior with socio-economic changes across the Neolithic and fill the gap between the Southern Levant and Anatolia. They also had adult human remains with suitable dentition for isotopic analyses and were from a geographically restricted area enabling Sr and O isotope baselines to be constructed and non-local individuals to be identified. To examine how mobility was linked to social and ritual practice, these data were integrated with evidence relating to age, sex and burial practice to understand the in-life and in-death identities of the individuals and investigate the connection to place and group membership in the earliest village societies of southwest Asia.

## Results

### Sr isotope analyses

**Fig. 2:**
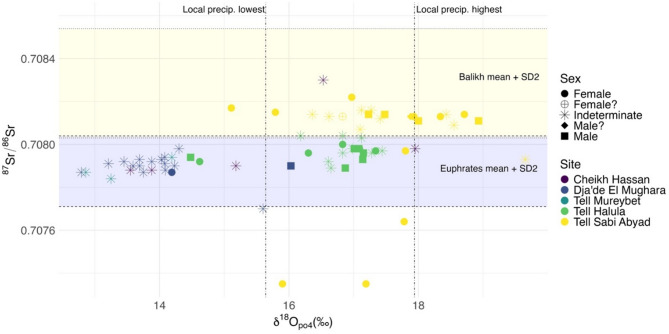
Scatterplot for all human enamel Sr and O isotope data by archeological site and sex. Dashed horizontal lines: Euphrates bioavailable Sr ratio mean and 2SD (area shaded blue). Dotted horizontal lines: Balikh bioavailable Sr ratio mean and 2SD (area shaded yellow). Dot-dash vertical lines: lowest and highest δ^18^O annual weighted means from local measurements of modern precipitation (see methods).

The tooth enamel ^87^Sr/^86^Sr ratios obtained from the 71 measured individuals range from 0.70735 to 0.70830 ($\overline{X}\pm SD=$ 0.70797 ± 0.00016) (Fig. 2, Supplementary Table S1). For descriptive statistics by site see Supplementary Table S2. Baselines of the local bioavailable Sr ratios were established using local modern plants and ancient human dentine (see methods, Supplementary Table S3) providing a range of 0.70771–0.70804 for the four Euphrates sites: Cheikh Hassan, Mureybet, Dja’de and Halula, and of 0.70803–0.70854 for the Balikh site: Sabi Abyad. The enamel ^87^Sr/^86^Sr ratios of most individuals fall within their local Sr baseline ranges, consistent with limited childhood or adult mobility, with just two of the 47 individuals from the Euphrates sites falling outside the local Sr baseline: the first, from Cheikh Hassan, yielding a higher value and the second, from Dja’de, a value just below the local range. Five individuals from Sabi Abyad have enamel ^87^Sr/^86^Sr ratios below the Balikh local Sr baseline.

### O stable isotope analyses

The phosphate δ^18^O_(v-smow)_ values of the 71 measured individuals cover a substantial range falling between 12.79 and 19.65‰ (per mille) (carbonate δ^18^O_(v-smow)_: 21.77 to 28.42‰) (Fig. 2, Supplementary Table S1). For descriptive statistics see Supplementary Table S4. Despite their close geographical proximity (Fig. 1), the δ^18^O_(v-smow)_ values from the different sites are significantly different after individuals with non-local ^87^Sr/^86^Sr ratios are removed (Kruskal Wallis test, H = 44.304, df = 4, *P* < 0.001). Individuals from the older sites (Cheikh Hassan, Dja’de, Mureybet) display significantly lower δ^18^O_(v-smow)_ values than those from younger sites (Halula, Sabi Abyad) (Mann–Whitney test, U = 6.011, df = 1, *P* < 0.001), and significant differences are observed between values from Dja’de and Halula, Dja’de and Sabi Abyad, and Mureybet and Sabi Abyad (Dunn-Bonferroni *post-hoc* pairwise comparisons, Supplementary Tables S5–S7). Although Cheikh Hassan yields an intermediate δ^18^O_(v-smow)_ mean value of 15.42‰ for the Euphrates sites, the individuals measured show considerable spread (13.55–17.95‰), with the two clustered values of Cheikh Hassan falling within the Dja’de and Mureybet ranges (Fig. 2). Sabi Abyad also shows considerable dispersion (15.11–18.93‰), suggesting the use of a wider range of environmental conditions or input of water sources compared to Dja’de, Mureybet and Halula (Supplementary Table S4).

**Fig. 3:**
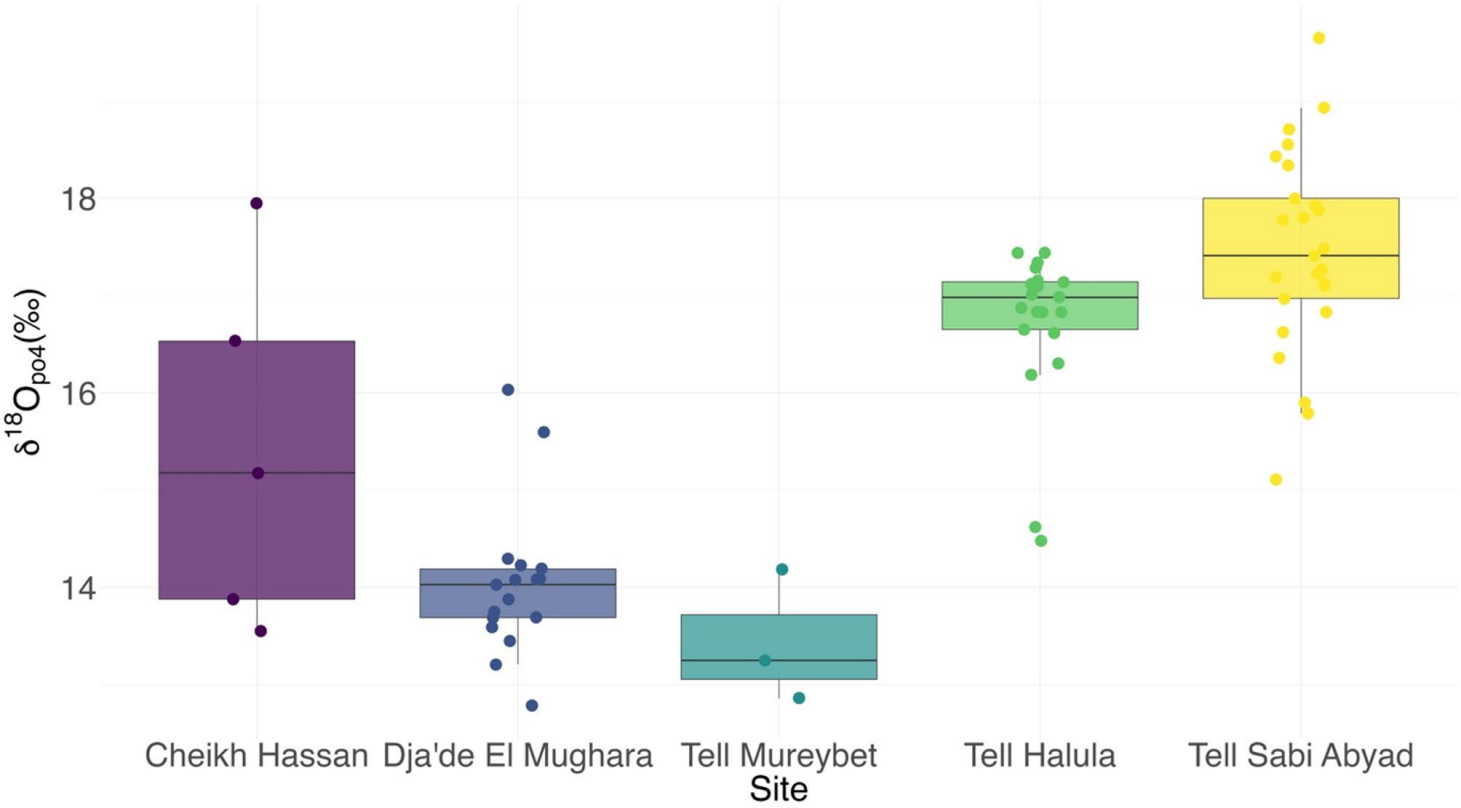
A box and whisker plot to show the distribution of phosphate oxygen isotope values across archeological sites in chronological order.

The interquartile range (IQR) at each site^39^ rather than modern rainfall data (see methods, Supplementary Notes) was used to identify possible non-locals due to a likely contribution of ^18^O-depleted meltwaters to the locally consumed waters during the Neolithic causing the low values observed at some of the sites (for an extended discussion see Supplementary Notes). The IQR was calculated for all sites except Mureybet, where samples were too few (Fig. 3). No outliers are identified at Cheikh Hassan possibly due to the small sample size combined with the relatively wide spread of these data. At Dja’de, three individuals fell outside of the IQR, one of which also had an ^87^Sr/^86^Sr ratio falling outside the local baseline. Another of these outliers is represented by a first molar (enamel formation: 4.5 months–4.5 years of age)^40^ and may have been impacted by breastfeeding (known to cause elevated values), thus not reliably indicating a non-local origin^30^. At Halula two outliers are found. Although one corresponds to a canine potentially affected by breastfeeding (enamel formation: 10.5 months to 6.5 years of age)^40^, the measured δ^18^O value is depleted rather than enriched (the expected result of breastfeeding) and thus indicates input from a different (presumably non-local) water source. Finally, at Sabi Abyad two individuals fall outside of the IQR, one of which also has a Sr ratio consistent with a non-local origin.

### The archeological context of the non-local individuals

A total of 11 possibly non-local individuals were identified in our study region from Sr ratios and/or O isotope values: Cheikh Hassan (1/5, 20%); Dja’de (2/17, 11.76%), Halula (2/22, 9.09%) and Sabi Abyad (6/25, 24%).

At Cheikh Hassan individual *T1, cranium 1* has an ^87^Sr/^86^Sr ratio consistent with a non-local origin. Context T1 comprised two adult crania of indeterminate sex, associated mandible fragments, and burnt material found in a pit^41^. In contrast, the other individual from this context (*T1, cranium 2*) an ^87^Sr/^86^Sr ratio consistent with a local origin. Although the small sample size and wide spread of the δ^18^O data at Cheikh Hassan does not allow for the identification of clear outliers, the values of both isolated crania are high compared to the other three individuals measured, all three of which are primary inhumations. Thus, although the Sr evidence of the two isolated crania does not suggest a common childhood origin, they both appear to have had input from different water sources than the individuals from primary inhumations at the site.

At Dja’de, individual *108-md a isolated,* of indeterminate sex, has a Sr ratio consistent with a non-local origin and an O isotope value falling outside of the IQR. This individual, represented by only the mandible, was found in Dépôt 108, as was another single mandible (individual *108-md c isolated),* which yielded Sr and O values compatible with local backgrounds (Supplementary Figs. S1a, S1b). Both were foundation offerings, buried in separate corners of the seventh phase of the so-called ‘house of the dead’, a building which contained the remains of 80 individuals^18,41^. A further 10 individuals from various phases of this building were also measured (Supplementary Table S1), and all display Sr ratios and O values consistent with the site location. Thus, the non-local individual *108-md a isolated* was afforded the same burial in this structure as others with a local origin. The other individual at Dja’de who may be of non-local origin based on a low δ^18^O value is a young adult of indeterminate sex (*283-sq C*) found in Dépôt 283, a context comprising five individuals buried near to one another outside Structure 147. This individual, also of unknown sex*,* appears to be a primary burial (Supplementary Fig. S2). No other individuals from this context were available for measurement.

At Halula, two individuals with outlying δ^18^O IQR values but Sr ratios within the local baseline are a young adult female (*H70*) and a young adult male (*H68a*). Notably, both were found in house DD (Supplementary Fig. S3), belonging to Occupation Phase 8 (end of the Middle PPNB). These possibly non-local individuals were afforded the same burial treatments as other individuals with values consistent with a local origin, including use of sub-floor inhumation in a seated position and the inclusion of similar burial goods. For example, *H70* (non-local) and *H136* (local) were both buried with stone beads, a stone tool, and marine shell belts^42,43^. That the two non-local individuals at Halula were buried together may suggest that elements of their shared life histories influenced where they were buried. However, the others buried in the same house sequence were local (*H69,* from the same phase, and from other phases *H30, H48, H49, H53,* and *H57*), indicating that other relational similarities associated with group membership between individuals, such as biological and/or fictive kinship, must have impacted their connection to the site and burial location.

At Sabi Abyad six individuals have ^87^Sr/^86^Sr ratios or δ^18^O values that are inconsistent with the local Sr baseline and/or the O isotope IQR for the site. Five of these are adult females (*BN08-028, BN08-053, BN09-011, BN09-035, BN09-040*) and one is an adolescent of indeterminate sex (*BN08-018)*. These individuals were buried in ways identical to local individuals, including non-normative burial treatment such as delayed burial, post-mortem manipulation, or elaborate burial assemblages, and/or burial in the same spatial clusters of the communal cemetery (Supplementary Fig. S4). A notable example is *BN08-053*, a non-local young adult female who was buried in the cemetery, phase Level B3-C8, in close spatial association with a local middle adult male (*BN08-056*). Both showed evidence of a highly elaborate and rare mortuary treatment involving the scorching of the inside of the thoracic cavity. They, however, had δ^15^N values that indicate different adult diets (8.3 vs. 10.1‰, respectively)^44^. Although these individuals had different life histories, potentially involving differential mobility patterns, they were buried together and afforded the same distinct treatment in death, highlighting the complexities of group membership.

## Discussion

The data presented here point to different levels and types of mobility from the PPNA to the LN in the Northern Levant. We now examine these data from a diachronic, inter-regional perspective, comparing Sr and O isotope and archeological evidence from other regions of southwest Asia. We consider both the frequency of inter-regional mobility in our dataset and more widely in southwest Asia as well as the permeability of social groups for new members in this region to address ideas of human attachment to place and group membership at this key point in human history.

**Fig. 4:**
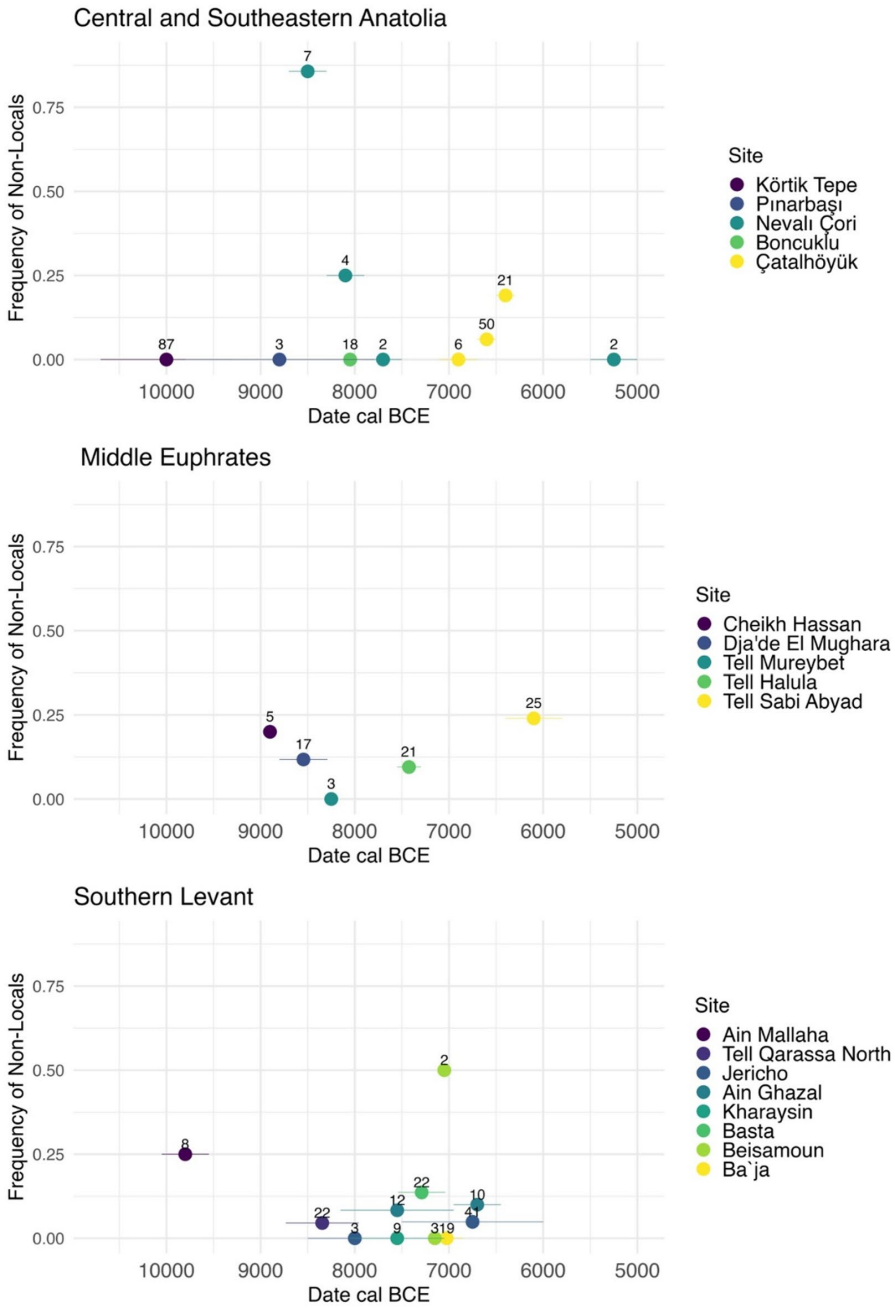
Frequency of non-local individuals across Epipaleolithic-Neolithic in southwest Asia by region and archeological site. Numbers above data points indicate study sample size. Non-local individuals were those identified by the authors of the studies. Data and references are available in Supplementary Table S8. One individual from Pınarbaşı (13,646–13,284 BCE) has been identified as a local and one individual from Ain Mallaha (13,050–11,050 BCE) has been identified as non-local but were not included in the plot because they are the only representatives for their time period.

The tendency towards more permanent and substantive architecture observed during the PPNA in the northern Levant^2,4^ signals a reduction in residential mobility between the 14th–8th millennia BCE, which is supported by the available Sr and O isotopic data elsewhere in southwest Asia. The Natufian sample in the Southern Levant is restricted to three sites, two of which have a considerable proportion of non-local individuals (Wadi Hammeh and ‘Ain Mahalla/Eynan), while in the third, Azraq 18, a small site with secondary burials, the two measured individuals are consistent with a local origin^35,45^ (Supplementary Table S8). Subsequently, the numbers of non-local individuals at sites decline substantially during the Neolithic (Fig. 4), which has been interpreted as evidence of increased sedentism and gradual transition towards more localized lifestyles^35^. Inferences about the timing of this process in different regions are, however, greatly limited by the scarcity of data. Temporal trends at certain sites such as Jericho (10th–end 7th millennium BCE) or Körtik Tepe (11th–10th millennium BCE) point to a decrease or a lack of evidence of mobility already at the start of the Neolithic, suggesting that at certain places highly locally focused lifestyles (i.e. sedentary or mobile in only a limited region) were already established during the PPNA, and in some regions, possibly also before the Neolithic^23^ (Fig. 4, Supplementary Table S8).

Isotopic evidence for PPNA Cheikh Hassan shows, however, a relatively broad distribution of δ^18^O values, and the Sr ratios suggest one non-local. Although the sample size is small, these data do suggest mobility in the PPNA that could have involved children accompanying adults on regular mobile activities, partner-exchange with neighboring regions, or individuals or parts of communities aggregating at specific sites. Archeological evidence at Cheikh Hassan points to a largely sedentary group that still exploited wild resources local to the area. Nonetheless, imported materials—such as central and east Anatolian obsidian—indicate (indirect) contact with more distant regions^24^. The mortuary record reveals both primary and secondary inhumations frequently associated with architectural structures^41,46^. This provides some evidence that in the PPNA forms of group membership associated with specific locations may have been taking shape. Although the inhabitants of the region did already invest in permanent architecture during this period^3^, childhood mobility and/or the building of group memberships in existing locations with non-locals likely occurred.

Architecture, subsistence strategies and ritual practice at PPNB sites in the region all point towards an increased attachment to place and group membership^4,15,16,47^, consistent with the available isotopic evidence in the Southern Levant and Central Anatolia of this period which suggests limited mobility. There is no evidence for mobility at PPNB Jericho^38^ and very limited mobility at Basta, Tell Qarassa, Beisamoun, Kharaysin and ‘Ain Ghazal^32,35^. Similarly, in Central Anatolia there is one possible non-local individual detected at Boncuklu (9th–8th millennium BCE)^23^. This contrasts with southeastern Anatolia, where isotopic evidence points at an overwhelming proportion of non-locals in the Early PPNB (early to mid-9th millennium BCE) at Nevalı Çori^37^ (Fig. 4, Supplementary Table S8).

The mortuary evidence at early PPNB Dja’de indicates an emphasis on spatial continuity with graves found in close association with architectural features, most notably the ‘house of the dead’^18,41,46^, but habitation was perhaps not fully sedentary based on depositional evidence for episodic occupation and the use of light structures in certain areas^17,48^. The small number of non-locals at Dja’de suggests that any mobile activities might have involved mostly adults or individuals not yet measured. The limited isotopic evidence for mobility also at Mureybet and Halula agrees with the archeological record indicating continuous and year-round habitation and evidence for animal domestication and plant management at middle PPNB Mureybet^41,49,50^ and at middle-late PPNB Halula a fully sedentary, agro-pastoral lifestyle^51,52^. At Halula it has also been proposed that the strictly regulated spatial placement of the dead within houses served to display and reinforce concepts of lineage, claim to place, and biological kinship or household membership^14,15^.

The limited levels of non-locals at PPNB sites suggest that demographic growth rather than population aggregation is a more likely reason for settlement size increase during this period in the Northern Levant, as proposed for the Southern Levant^35^ and central Anatolia^23^. The presence of imports (e.g. Anatolian obsidian) demonstrates that the PPNB populations of the Northern Levant were in contact with other regions of southwest Asia either directly or through exchange networks^25,26^. Whole genome evidence also points at interregional gene flow from the onset of agriculture across southwest Asia, evidenced in the western side of the Northern Levant/Upper Mesopotamia by a composite of Anatolian, Central Zagros and Southern Levantine Neolithic genetic affinities^22,53^. Since the isotopic data is compatible with limited interregional population mobility and/or few non-locals, much of this observed interregional connectivity may have been facilitated through highly localized networks, i.e. within the Middle Euphrates, and/or through longer-distance logistical mobility of small numbers of adult individuals, which would be undetectable through isotopic methods. Permeability of communities to other groups, evidenced by the burial of non-local individuals alongside locals, could have facilitated gene-flow.

By the LN the agropastoral way of life was firmly established in northern Syria and more mobile forms of pastoralism appear to have gained in importance, accompanied with new forms of interregional connectivity, for example evidenced by the widespread appearance of shared decorated pottery styles^54^. The Sr and O isotopic evidence at Sabi Abyad also suggests a departure from the apparent emphasis on localized logistical and/or residential behaviors characterizing the PPNB and indicating instead that the archeological evidence of inter-regionality during the LN was accompanied by the movement of people. Although most individuals at Sabi Abyad yielded Sr isotope ratios in line with the locally expected baselines, consistent with a broadly local origin and limited interregional childhood residential or logistical mobility, the greater numbers of non-locals compared to the PPN periods are consistent with a more diverse population with varied mobility patterns and/or geographic origins. Carbon and nitrogen stable isotope analyses of human remains at Tell Sabi Abyad also show increasing variability over time^44^. Interestingly, domestic caprine isotope data from the site show an opposite pattern, tentatively interpreted as being the result of the pooling of flocks to facilitate mobile herding in the steppes^44^. The increase in human mobility in the LN of the Northern Levant accords with evidence from LN Central Anatolia^23^ where an increase in human mobility during the middle phase of occupation at Çatalhöyük occurred, when the population level peaked. The mobility at Çatalhöyük continued into later phases, which were associated with a reduction in population size and settlement density (Fig. 4, Supplementary Table S8).

Genetic evidence from Central Anatolia suggests that biological relatedness decreased from the PPNB to the LN between those buried in houses^21^. Accompanied by the rising number of non-locals, this suggests increasing flexibility and variability in kinship arrangements and co-residence^23^. At Sabi Abyad, partly contemporaneous with Çatalhöyük, the architectural and burial evidence also suggests decreased emphasis on spatial permanence and house-based identity^19,44^. This suggests, when combined with the isotopic evidence for mobility, that in the Northern Levant community boundaries became more permeable during the later phases of the Neolithic. It is not clear whether this trend is part of a Levantine or southwest Asian pattern more broadly as there are currently no Sr or O isotope data for the Southern Levant LN. Genetic evidence indicates that, by the LN, Central Anatolian populations experienced increased gene flow from Upper Mesopotamian groups with additional admixture from a Neolithic Levantine source^22,53,55^. This highlights the increasing population connectedness throughout the LN, suggesting that future isotopic work in the Southern Levant on later Neolithic sites may also reveal increased evidence for non-locals.

The apparent shifts in mobility detected over time in our dataset have profound social implications for group membership and community integration. The isotopic evidence suggests that being non-local or highly mobile in childhood had little to no impact on the burial treatment afforded to individuals at the sites in our study region. For example, both secondary and primary burials are represented amongst the non-locals identified in our data set, as amongst those with local values. Elsewhere, inclusive burial practices for non-locals appear to have been a widespread phenomenon. At Çatalhöyük^23^, Nevalı Çori^37^, and Ba’ja^36^ there were no observable associations between non-local isotopic signatures and non-normative treatment in death, even at sites with a large variability in mortuary treatment amongst the individuals analyzed. Only at ‘Ain Mallaha/Eynan and Beisamoun were some non-locals differentiated from the rest of the community through burial practices^35^. Although examples exist of the correlation of specific mortuary treatments with archeologically observable in-life traits such as age, sex, diet, health and bodily modification^23,56^, these are exceptions. More often such correlations are absent. This suggests that the mortuary sphere was not commonly or consistently used to display in-life identities, but perhaps to actively downplay intra-communal differences. Individuals with variable backgrounds were not only afforded the same initial, basic burial rites, but also actively commemorated in the same ways, perhaps signaling inclusivity towards more mobile individuals and encouraging social cohesion.

Group membership may also have been impacted by partner-exchange strategies. Two possible non-locals at Halula were identified as a male and a female, indicating that both sexes were mobile. Unfortunately, the small proportions of non-locals in the other PPN populations and/or indeterminate sex of many of the sampled individuals prevents discussion of endogamous or exogamous practices more generally. An absence of clear-cut patterns is also observed elsewhere in the PPN of southwest Asia, although some sites have yielded evidence for endogamy versus exogamy and matrilocality versus patrilocality. Matrilocality has been tentatively proposed at Kfar HaHoresh^57^. Endogamic practices have been suggested for Basta^32^ and the lack of non-locals at either Körtik Tepe or Jericho could suggest endogamous practices operated at those sites too^33,38^. In the LN, at Sabi Abyad five out of six non-locals were female while the sixth was an adolescent of indeterminate sex. Considering that the enamel sampled in these individuals reflects childhood to adolescence, this suggests that either female children were more commonly involved in mobile activities, or that females were more likely than males to join the Sabi Abyad community later in life. The latter, female residential mobility, could point to the existence of patrilocal traditions in the LN of the Northern Levant, although other reasons for residential and/or logistical mobility such as socioeconomic or political tasks should not be excluded. At Çatalhöyük, both males and females were mobile on a supra-local scale^23^. On the site-level, even though a lack of matrilineal relationships within co-burials was originally observed^58^, this pattern was recently reconsidered with the analysis of more individuals^59^. This study showed that, although inter-site mobility was not sex biased, the maternal lineage often connected household members. The implication that at Çatalhöyük women often remained tied to their maternal house (but not necessarily to their settlement), suggests LN mobility practices were highly complex, both within and amongst communities.

In summary, the diversity in the mobility practices of Neolithic populations of the Northern Levant accords well with what is observed for the period across southwest Asia, where various forms of mobility practices independent of site size and economic practice have been evidenced^7,23,32,33,35–38^. There is, however, a broader trend that is apparent within the PPN evidence. The transition between the PPNA and PPNB marks substantial changes in the new ways Neolithic people were living, many of which tied more people to places in the landscape^1–4^. Domestic plants and animals were increasingly becoming part of the food procurement strategies, populations and settlement sizes were expanding, and ritual practices flourished^5,6,8,60^. In particular, the burial practices of the PPNB, although originating in the PPNA/Natufian and showing continuity into the LN, are among the most elaborate and varied witnessed across southwest Asia and have been repeatedly identified as critical for encouraging social cohesion^9,10^. The lower levels of PPNB mobility against a backdrop of such burial practices suggest that communities were focused on maintaining relationships within the group and residing in relatively fixed locations by ensuring that mobility and partner-exchange were either intra-site or highly localized. This may have been partly how PPNB communities of southwest Asia consolidated their relationships with one another. The mobility that emerges in the LN,the evidence of which is still lacking for the Southern Levant, suggests that mobility shifted from highly localized to increasingly regional, supplementing, reinforcing or expanding existing exchange and/or reproductive networks. Non-local or highly mobile individuals’ ties to new communities were strengthened by burial practices that sought to reinforce group and community membership even beyond the PPNB and new ways of living in the later Neolithic.

## Methods

### Sampling

A total of 71 individuals from five sites in northern Syria (Figs. 1, 2, Supplementary Table S1), spanning the 9th to the 6th millennia BCE (late PPNA to LN), were sampled for Sr and O isotope analyses. Due to sampling constraints, including the availability of suitable samples and the need to minimize destructive sampling of this rare material, only one tooth per individual was sampled. Permanent adult teeth with minimal wear and no evidence for caries or enamel defects were prioritized. This sampling strategy resulted in the following totals: Cheikh Hassan (n = 5), Dja’de (n = 17), Mureybet (n = 3), Halula (n = 21) and Sabi Abyad (n = 25). Most (n = 38) were M2 teeth (enamel formation: 2.5–8.5 years), others included M3 (n = 10) (enamel formation: 8.5–14.5 years), P1/P2 (n = 18) (enamel formation: 2.5–8.5 years), M1 (n = 5) (enamel formation: 4.5 months to 4.5 years), C (n = 1) (enamel formation: 10.5 months to 6.5 years). Although enamel formation of M1 and C begins during breastfeeding, which can impact Sr and O isotope values^61^, they were occasionally included to increase sample-size and taken into consideration in data interpretation. All tooth development ages follow established criteria^40^.

The selected teeth were sampled following standard Durham University laboratory protocols at the Professor Elizabeth Slater Archaeological Research Laboratories, University of Liverpool. All tools were cleaned in an ultrasonic bath, rinsed with ultrapure water, and dried with acetone. The exterior of each tooth was cleaned and abraded using a tungsten carbide rosebud bur attached to a hand-held Dremel drill. The crown was then bisected using a diamond-coated blade. Slices of enamel for Sr analysis were cut lengthwise from the least worn/damaged half using a sterile blade. The dentine was removed using a rosebud bur with small chunks (10–15 mg) reserved for Sr baseline analysis. Small enamel chips from the same half were used for O isotope analysis and were crushed to a fine powder using an agate pestle and mortar.

### Sr isotope analysis

Enamel samples (c. 20 mg) were prepared for Sr isotope analysis using established methods^62^ at the Arthur Holmes Isotope Geology Laboratory (AHIGL), Durham University. In brief, samples were digested overnight in 3M HNO_3_ on a hotplate at 100 °C before being loaded onto cleaned and preconditioned columns containing Eichrom Sr-specific resin. A purified Sr fraction was eluted from the column in 500 µL H_2_O and acidified with 15.5M HNO_3_ to yield a 3% HNO_3_ solution. The size of the ^86^Sr beam was tested for each sample to derive a dilution factor so that each sample yielded a beam size of approximately 25V ^88^Sr to match the intensity of the isotopic reference material, NBS987. Samples were aspirated using an ESI PFA-50 nebulizer coupled to a Glass Expansion Cinnabar micro-cyclonic spray chamber. Sr isotopes were measured using a static multi-collection routine with each measurement comprising a single block of 50 cycles with an integration time of 4s per cycle (total analysis time ~ 3.5min). Instrumental mass bias was corrected for using an ^88^Sr/^86^Sr ratio of 8.375209 (the reciprocal of the more commonly used ^86^Sr/^88^Sr ratio of 0.1194) and an exponential law. Corrections for isobaric interferences from Rb and Kr on ^87^Sr and ^86^Sr were performed using ^85^Rb and ^83^Kr as the monitor masses but were insignificant. In all samples the ^85^Rb intensity was < 18.3 mV with an ^85^Rb/^88^Sr ratio of ≤ 0.32 (average 0.02) in the first run and of ≤ 0.7 (average 0.05) in the second run. ^83^Kr was between 0.26 and 0.32 mV in all samples. Samples were measured during two analytical sessions during which the average ^87^Sr/^86^Sr ratio and reproducibility for the international isotope reference material NBS987 was 0.710252 ± 0.000012 (2σ; n = 14) in the first run and 0.710267 ± 0.000006 (2σ; n = 11). Maximum error based on internal precision of individual analysis and analytical reproducibility of the reference material is 0.000012 (2σ). Sr isotope data for samples was normalized to an accepted value for NBS987 of 0.71024.

### O isotope analysis

The powdered enamel for O stable isotope analysis was transferred to the Stable Isotope Laboratory, Earth Sciences Department, Durham University. O (δ^18^O) isotope ratios were measured in the carbonate (CO_3_) component of tooth enamel. For each tooth, approximately 2.5 mg of powdered sample was weighed and transferred into individual exetainer vials. Vials were then flushed with helium (99.999%, grade N5.0) and CO_2_ was liberated by reaction with 99% ortho-phosphoric acid for 2 h at 72 °C. The resultant gas mix of helium and CO_2_ was transferred through a Thermo Fisher Scientific Gasbench II in which a gas chromatographic column separated the CO_2_ from the gas mixture and then passed into a Thermo Fisher Scientific MAT 253 gas source mass spectrometer for isotopic analysis.

The following international reference materials were analyzed within each batch of samples: NBS 18 (calcite, n = 3), IAEA-CO-1 (marble, n = 3) and LSVEC (lithium carbonate, n = 3). In addition, two internal standards: DCS01 (calcium carbonate, n = 6) and Dobbins (horse tooth, n = 2) were also analyzed. Repeated analysis of both international and internal standards yielded an analytical precision better than 0.1‰ (2SD) for δ^18^O. Duplicate sample analysis yielded precision with a mean difference of 0.2‰ (2SD) for δ^18^O (n = 72). Normalizations and corrections were made using IAEA-CO-1 and LSVEC and δ^18^O values reported relative to the Vienna PeeDee Belemnite (VPDB) standard. δ^18^O was additionally reported relative to the Vienna Standard Mean Ocean Water (VSMOW) standard for comparison purposes. Human δ^18^O_carbonate_ values were subsequently converted to δ^18^O_phosphate_ values^63^.

### Establishing the local Sr baseline

**Fig. 5:**
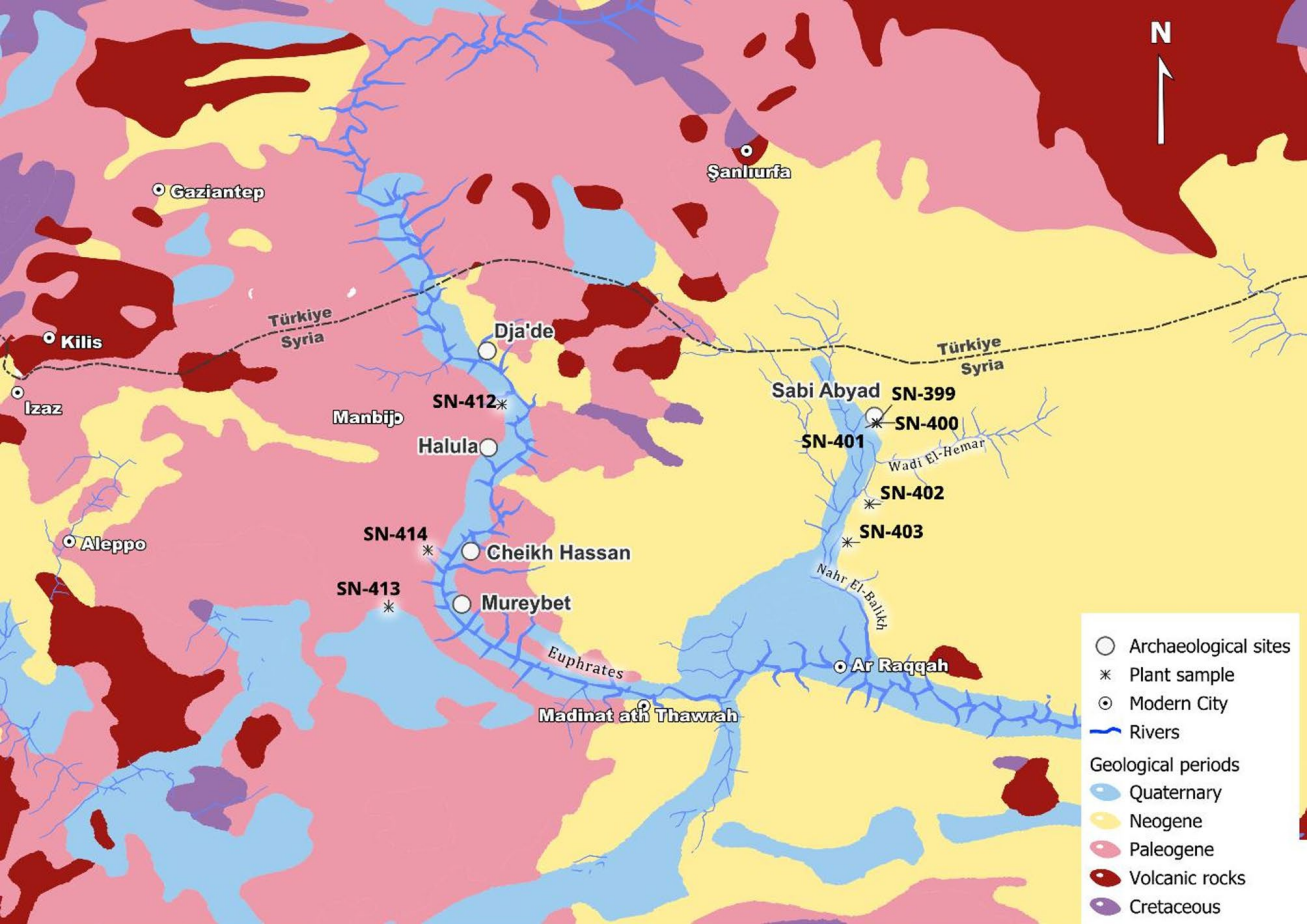
Simplified geological map of the study area. Locations of the major geological substrates, water courses, study sites, and modern plants sampled for the Sr baseline (Supplementary Table S3). Blue: Quaternary alluvial deposits; Yellow: Neogene sediments; Pink: Paleogene sediments; Red: Quaternary and Neogene basalts; Purple: Cretaceous sediments. Map based on Syrian Arab Republic Ministry of Petroleum and Mineral Resources Geological Map of Syria, 1986 (scale: 1:1.000.000) and the Turkish General Directorate of Mineral Research and Exploration Geological Map of Turkey, sheets: Hatay and Diyarbakır, 2002 (scale 1:500.000).

To identify non-local individuals, we established a Sr baseline reflecting the local geology and the bioavailable ^87^Sr/^86^Sr ratios at each site. Cheikh Hassan, Dja’de, Mureybet, Halula and Sabi Abyad, are located on the Neogene and Paleogene sediments of inland Syria, with Miocene Lagoonal deposits in the Jazirah, surrounded by Paleocene sediments^64^. Cheikh Hassan, Dja’de, Mureybet, and Halula are located along the Euphrates, which in Türkiye flows through Mesozoic metamorphic, Tertiary volcano sedimentary rocks, Eocene limestones and Plio—Quaternary alluvial basin, followed by Paleogene marl and marly limestone in northern Syria^65^. Sabi Abyad is located on the Quaternary floodplains of the Balikh flanked by a relatively homogenous geology dominated by Neogene rocks, those of the upper Syrian Euphrates are surrounded by a patchwork of Neogene, Paleogene and Cretaceous rocks^66^ (Fig. 5).

**Fig. 6:**
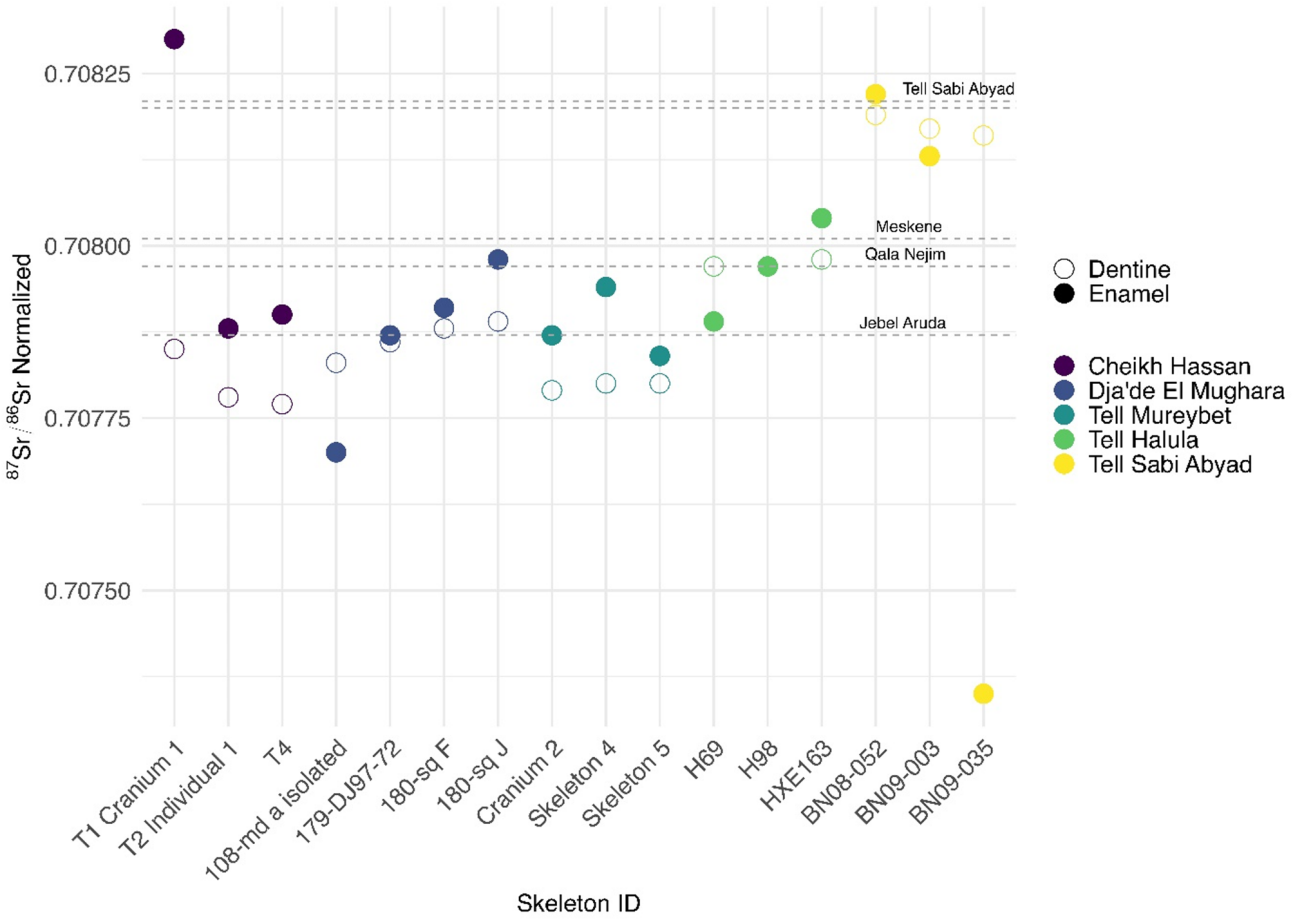
Local bioavailable Sr. Dentine samples: open circles, enamel values: closed circles. Open and closed circles in the same vertical position indicate the same tooth was used. Local plants: dashed lines. See Supplementary Table S3 for the data used to establish the baselines.

We used published^67^ and unpublished data from 12 modern plants and measured 16 human dentine samples from the same teeth sampled for enamel to map the Euphrates and Balikh baseline for our sites (Figs. 1, 5, 6, Supplementary Table S3). Plant samples collected by the Sabi Abyad team were prepared for Sr isotope analysis using standard column chemistry methods at the FALW Vrije Universiteit Amsterdam in 2009. In brief, 0.5 ml 3.0 N HNO_3_ sample solutions were loaded onto cleaned and preconditioned columns (0.085 ml quartz column, filled with Sr-resin medium [100–150 μm]). A purified Sr fraction was eluted from the column in 0.8 ml milli-Q water [or 0.05 N HNO_3_ dist.], dried down overnight at 120 °C, and nitrated with HNO_3_ dist. Subsequently, 1µl of 3N HNO_3_ was added to yield a sample solution. All measurements were performed on a Finnigan MAT 262 mass spectrometer and measured against the international standard NBS987.

Since modern plant ^87^Sr/^86^Sr ratios can be affected by sediment contributions from aeolian or fluvial deposits, fertilizers and pollution, the human dentine, which takes up Sr from the burial environment, was used to confirm the expected local values^68^. It must be noted that the use of dentine ^87^Sr/^86^Sr ratios to create regional strontium isotope baselines is limited by uncertainties in establishing whether the dentine in the sample set has been fully diagenetically altered to the point that it accurately reflects the local ^87^Sr/^86^Sr of the burial environment. Nonetheless, its use in the current study in conjunction with modern plant ^87^Sr/^86^Sr ratios represents a viable option, especially in the absence of other materials.

After the ^87^Sr/^86^Sr ratios of the tooth enamel were measured, we selected individuals yielding the highest, middle, and lowest ^87^Sr/^86^Sr ratios of each site (where sufficient material was available) for dentine baseline analysis. This sampling strategy targeted both local and non-local individuals as determined by the Sr evidence from enamel. Based on three modern plants (collected at Qala Neijm, Jebel Aruda and Meskene) and 13 dentine samples from Dja’de, Halula, Cheikh Hassan and Mureybet, the bioavailable local Sr signature of the Middle Euphrates was estimated to be 0.70771–0.70804 (Supplementary Table S3). Using five modern plants and three human dentine samples collected at Sabi Abyad and other locations along the Balikh (Fig. 5) and published data^67^ the local baseline range of the Balikh was estimated to be 0.70803–0.70854.

Figure 6 and Supplementary Table S3 show the dentine ^87^Sr/^86^Sr ratio range for each site falls within a much more restricted range compared to those of the enamel. Although the dentine ratios ‘track’ their paired enamel ratio slightly, suggesting post-depositional uptake from the burial environment is not complete, they cluster well with the other dentine measurements from their respective sites and with measured plants from the region. The clustering of the dentine samples (per site and in more general terms with the local plant data) confirms the viability of the use of dentine for local baseline purposes in this region. Different dentine and enamel ^87^Sr/^86^Sr ratios also suggest the enamel has not been affected by diagenesis and subject to post-depositional uptake of soil Sr.

### Establishing locally expected O values

The isotopic composition of modern rainfall, surface water and groundwater can be used to understand past interregional variability in δ^18^O values^69^. However, since climatological and hydrological changes can affect the O isotope values in water sources, modern values provide only an approximation of possible past local values. When all modern evidence in the study region is combined (see Supplementary Notes, Supplementary Table S9), individuals from our sites would be expected to have consumed water with a δ^18^O_mw_ value between − 9.19 and − 4.86‰, thus producing phosphate δ^18^O_(v-smow)_ values in human tissues ranging between 15.64 and 17.94‰, and potentially somewhat higher values due to the potential impact of cultural behaviors such boiling, stewing, brewing, and milk consumption discussed earlier.

Whereas the majority of individuals from Halula and Sabi Abyad yield δ^18^O values compatible with the locally expected range, the values of some individuals from Cheikh Hassan, Dja’de, Mureybet and Halula (individuals that measured c. 12–15‰) are unexpectedly low for the region, falling below the lowest observed annual weighted means anywhere in Syria and southeastern Anatolia (Supplementary Notes, Supplementary Table S9). Currently, the most likely explanation for the discrepancy between the modern rainfall data and the δ^18^O values from much of our human tooth enamel is the contribution of ^18^O-depleted melt waters introduced into the headwaters of the Euphrates in Türkiye during the Neolithic (see Supplementary Notes for an extended discussion).

In addition to the likely input of water sources which have changed in isotopic composition since the Neolithic, the large variations of δ^18^O values within any given population caused by physiological and/or cultural factors mean that statistical methods which identify outliers in the sample per site, rather than modern water isotope data alone, are better suited to identify mobile individuals. Robust measures of scale such as the interquartile range (IQR) are most appropriate for such purposes^33^ and were applied to all our sites, except for Mureybet, which only had three data points.

### Statistics and data plots

Statistical analyses were conducted with IBM SPSS statistics (version 29.0.2.0). Basic statistics (mean, CI mean, standard error (SE), standard deviation (SD), variance, median, minimum and maximum values and range) were computed for the Sr and O isotopic data to obtain an overview of their distribution across sites and periods (Supplementary Tables S2, S4). The normality of the distribution of the δ^18^O data across sites and periods once Sr outliers were removed was assessed using the Shapiro–Wilk test. As the data was not normally distributed (Supplementary Tables S5, S6), non-parametric Kruskal–Wallis and Mann–Whitney tests were used to assess the differences in δ^18^O values across sites and periods respectively. Dunn-Bonferroni *post-hoc* pairwise test was applied to examine differences in δ^18^O values between each pair of sites (Supplementary Table S7). All plots were produced using R Statistical Software (v4.3.2)^70^ in RStudio (v2023.12.0.369)^71^. Input data were manipulated using the readr v2.1.5 and deplyr v1.1.4 packages^72,73^, and color-blind friendly plots were generated using the ggplot2 v3.4.4, patchwork v 1.2.0, cowplot v1.1.3, and viridis v0.6.4 packages^74–77^. Code used to generate the plots can be found at https://github.com/Kelzor/Plug-et-al-Neolithic-residential-mobility-plots.

### Permissions and approval

All experiments were performed in accordance with guidelines and regulations from the leading institution (Durham University). Experimental protocols were approved by the ethics committee of the Department of Archaeology, Durham University, where all experiments were conducted (Application Number ARCH-2019-06-24T15_51_36-kvgm94). Informed consent is not applicable in this case, as the samples belonged to prehistoric individuals that lived 9000–7000 years ago. Permission to analyze these archaeological samples was obtained from the relevant authorities, in particular the Syrian Directorate-General of Antiquities and Museums (DGAM), and sample depositaries.

## Supplementary Information

Supplementary Material 1.

Supplementary Material 2.

## Data Availability

All data are available in the main text or the supplementary materials.
