# Supplementary material for: Strontium and oxygen isotope analysis reveals changing connections to place and group membership in the world’s earliest village societies
Source: Sci Rep. 2025 Oct 3;15:34598. doi: 10.1038/s41598-025-18134-3 (PMC12494774; doi:10.1038/s41598-025-18134-3)
Supplement: Supplementary file 1 — Supplementary Material 1. [file 41598_2025_18134_MOESM1_ESM.docx]

**SUPPLEMENTARY MATERIALS**

**Strontium and Oxygen Isotope Analysis Reveals Changing Connections to Place and Group Membership in the World’s Earliest Village Societies**

Plug et al.

(corresponding author Jessica.Pearson@liverpool.ac.uk)

# **THIS PDF FILE INCLUDES:**

Supplementary Notes

Supplementary Figures S1 to S4

*Supplementary Tables S1 to S9 are provided as a separate .xlsx file.*

# **SUPPLEMENTARY NOTES**

## **Site descriptions**

#### Cheikh Hassan

Cheikh Hassan is located on the east bank of the Euphrates but is currently submerged by the Tabqa dam. The size of the site is unclear since it was completely covered by a later Bronze Age tell. Originally excavated by Jacques Cauvin in the 1970s, and later by Johannes Boese, followed by Danielle Stordeur, it was occupied from the 9^th^ to early 8^th^ millennium BCE, representing the PPNA to the earliest PPNB in the region [1]. The late PPNA habitation is argued to be a largely sedentary community of hunter-gatherers living in small rectilinear houses, representing an early transition from oval to rectilinear buildings that took place across southwest Asia.

The zooarchaeological evidence shows the exploitation of a range of wild animals from the riverine forest and steppe, which during the PPNA focused on equids, gazelles, and to a lesser extent aurochs [2]. Waterfowl and steppe birds were also hunted. Exchange networks are evidenced by imported materials, including central and east Anatolian obsidian [3], which are present in small amounts relative to local flint.

The mortuary record of the site comprises nine contexts (eight in the northern area and one in the southern area) dating to the late PPNA (~8900 BCE), including both primary and secondary inhumations. Most are associated with architectural structures [1] and with animal bones, chipped stone and burnt material. The five individuals sampled were chosen as these had suitable teeth for measurement and represent both primary and secondary burial practices.

#### Dja'de el-Mughara

Dja’de, around 1.5 ha in size, is located on a terrace on the east bank of the Euphrates at the edge of the Jezireh steppe. It was occupied during the 9^th^ millennium BCE (late PPNA to early PPNB), followed by a second occupation in the 7^th^ millennium BCE. Excavations were directed by Eric Coqueugniot. The architecture at the site comprises rectilinear buildings with stone walls and floors, circular buildings and light structures [4]. In the PPNA phase a semi-subterranean and circular building (7.5 m in diameter) was decorated with a polychrome geometric pattern with internal dividers separating the space and has been interpreted as a likely communal building. Other monumental architecture includes a substantial 1.1-0.7 m thick wall possibly of symbolic purpose. Grooved stone and engraved slabs have also been found among the material culture dominated by chipped stone and bone tools.

In the early PPNB, the buildings shift from circular to rectilinear, multi-roomed structures, sometimes with pebbled floors. In one of the PPNB subphases butchery waste was recovered over a large area with analysis showing species largely limited to aurochs and equids. Bone reduction, presumably for marrow and grease extraction was high, with surface modifications of the bone suggesting rapid deposition. This was interpretated as a processing and feasting deposit operating at communal level rather than the usual household level due to the high volume of material deposited. During the second phase of the PPNB, architecture shifts to smaller, unicellular rectangular buildings separated by exterior spaces. Evidence for episodic habitation and light structures (represented by fire spots and postholes) in certain areas of the site may indicate seasonal occupation [4].

In addition to various wild fruits, edible plant species include several wild cereals and legumes, with some evidence of pre-domestic cultivation [5,6]. The animal assemblage of the earliest PPNB phase is dominated by wild gazelle, followed by equids and then cattle [7]. Wild avian resources such as bustards and raptors were also found. The presence of dogs and evidence for morphological changes in the cattle suggest early steps towards animal domestication.

A total of 34 mortuary contexts containing at least 116 individuals were excavated, most of which date to the early PPNB [5]. The burial practices were diverse, which is a hallmark of the PPNB. Both primary and secondary inhumations were represented as well as single and multiple deposits [1]. Due to their association with large numbers of human remains, certain buildings show a particular mortuary relevance, including the so-called ‘house of the dead’. This structure appears to have been rebuilt on the same footprint seven times over a period of 250-300 years during which at least 80 individuals were buried [1,8]. A total of 17 individuals from the early PPNB, including 12 from the ‘house of the dead’, were sampled (Supplementary Figs S1a, S1b and S2).

#### Tell Mureybet

Mureybet is 0.5 ha in size and located on the east bank of the Euphrates, submerged after the construction of the Tabqa dam. It was excavated in the 1960s by Maurits Van Loon, followed by Jacques Cauvin. The site was settled at the end of the 11^th^ millennium BCE, during the Natufian period, and was inhabited throughout the PPNA and part of the PPNB, until the late 9^th^ millennium BCE. During the PPN, the site appears to have been occupied continuously and year-round [1]. The long site history shows the local transition from a nomadic hunting and gathering lifestyle to early settled village life, as well as the introduction of communal buildings [9] and a transition from round, single-roomed to rectangular, multi-roomed structures that potentially reflect changes in social behaviors and/or storage practices.

Inhabitants made use of several natural resources, including riverine and steppe environments as well as a nearby flint source [10,11]. The zooarchaeological, and to a lesser extent the archaeobotanical record [6], shows gradual steps towards the management of wild species, and eventually domestication in the middle PPNB [11]. The animal species exploited were diverse and included many mammals but also tens of species of birds, reptiles, fish and mollusks. Wild cereals of wheat, barley and rye were collected as well as wild lentils, vetches, legumes, fruit and nuts.

Ten PPNB mortuary deposits were found at Mureybet, including primary and secondary deposits, often associated with domestic structures [1]. Three individuals, dating to the middle PPNB (8300-8200 BCE) were sampled.

#### Tell Halula

Halula, a relatively large ~8 ha site, is located on the west bank of the Euphrates with access to riverine and steppe environments. The site, excavated by Miquel Molist and team since the early ‘90s, was inhabited from the early 8^th^ until the mid-6^th^ millennium BCE, representing the middle PPNB to Halaf periods [12,13]. From the earliest phases, the plant and animal economy were dominated by fully domesticated species [14–16], although natural resources continued to be exploited. For the PPN periods, the archaeological evidence points to a fully sedentary lifestyle with year-round occupation. Extra-regional connections, particularly with Anatolia, are evident through the presence of imported raw materials and finished goods [17].

The architecture is dominated by highly standardized, rectangular domestic structures. Underneath the floors of these houses, which often comprised sequences of buildings constructed on the same footprint over several generations, the remains of more than 130 individuals were found [18]. It appears that, as with the architectural practice, mortuary behavior was guided by strong standardization. Although much variability in the material funerary assemblage existed [17], virtually all human remains recovered at the site came from single, primary, sub-floor inhumations in an upright-seated position. It has been argued that the strictly regulated spatial placement of the dead served to highlight and reinforce concepts of lineage and familiar structure [19]. A total of 21 individuals dating to the middle to late PPNB (7550-7300 BCE) phases of the site were sampled.

#### Tell Sabi Abyad

Sabi Abyad, a site comprising four mounds, is located along the northern part of the Balikh, a perennial tributary of the Euphrates which is flanked by the steppe plains. Excavations at the site, directed by Peter Akkermans from the late 80s until 2010, focused on the Neolithic and Late Bronze Age habitation on the largest mound, which is approximately 5 ha in size. The Neolithic habitation at this mound, spanning the 7^th^ and early 6^th^ millennia BCE (thus representing the Initial Pottery Neolithic to the Halaf), is marked by several spatially distinct areas of habitation, each comprising modest numbers of multi-roomed, rectilinear structures of varying sizes [20–23]. From the late 7^th^ millennium onward, such buildings are accompanied by circular structures characteristic of the Halaf, known as ‘tholoi’.

The location of the site would have been well-watered, providing good grazing for livestock (i.e. domesticated sheep, goat, cattle and pigs) and favorable conditions for crop cultivation, primarily domesticated cereals and legumes. Wild resources were also exploited. In the late 7^th^ and early 6^th^ millennium, increasingly mobile forms of animal herding have been proposed [24,25], hypothesized as having been facilitated by a bimodal population of sedentary agriculturalists and mobile pastoralists [26,27]. The adoption of pottery vessels is apparent at the start at the 7^th^ millennium BCE [22,28], and the gradual, local development of the characteristic decorated pottery styles found across southwest Asia during the later 7^th^ and 6^th^ millennia BCE are suggestive of new forms of interregional connectivity [29,30].

The mortuary record includes the remains of over 300 individuals, most of which were found in cemetery areas dating to the mid-late 7^th^ to early 6^th^ millennium BCE [31,32]. Mortuary behavior at the site is diverse, including primary and secondary mortuary treatments, a wide range of burial goods, as well as single, multiple, and collective inhumation [32,33]. A total of 25 PN individuals were sampled in the context of this study.

## **Modern rainfall data**

Altitudinal effects are one of the main factors behind the local variability in modern rainwater δ^18^O values in Syria, with higher altitudes yielding lower δ^18^O values with a -0.18‰ shift per 100 m elevation [34]. A temperature effect is shown by an average δ^18^O gradient of 0.34 ± 0.03‰ per ◦C [34] and evaporation from modern irrigation and damming of the Euphrates also has a measurable impact on river δ^18^O values [35].

The Global Network of Isotopes in Precipitation (GNIP) provides data from 14 weather stations in Syria, with those at Raqqa, Aleppo and Jarablous located closest to our sites. The annual weighted means, which should be the most comparable to bulk enamel, from these three stations were checked against the Online Isotopes in Precipitation Calculator (OIPC), which estimates δ^18^O values specific to geographical locations, and found to agree. In addition to rainwater, rivers and groundwater are likely sources of drinking water. The Global Network of Isotopes in Rivers (GNIR) provides water isotope measurements at three locations along the Middle Euphrates (Jarablous, Qaraqousak and Maskaneh) and one along the Balikh (Raqqa), yielding values similar to the local precipitation, although those of the Balikh are on the higher end of the range. These rainfall and river water δ^18^O values were converted to δ^18^O_(v-smow)_ [36] and summarized in Supplementary Table S9. There is some variability in the δ^18^O values of underground water sources in Syria, as is the case in most regions [37], but these are generally consistent with the local precipitation values [38].

When all modern evidence in the study region is combined (see Table S9), individuals from our sites would be expected to have consumed water with a δ^18^O_mw_ value between -9.19 and -4.86‰, thus producing phosphate δ^18^O_(v-smow)_ values in human tissues ranging between 15.64 and 17.94‰. While measurements of modern precipitation to the north of the study region at Diyarbakır, Adana, Erdemli and Güzeloluk range between δ^18^O_mw_ -12.07 and -3.45‰ (δ^18^O_(v-smow)_ 14.11-18.69‰) these appear to be attributed to a single year at Diyarbakır with an unusually low δ^18^O annual weighted mean value from Global Network of Isotopes in Precipitation (GNIP) database.

## **Interpreting the low δ^18^O values at the Euphrates sites**

A substantial proportion of the individuals found at the Euphrates sites (Cheikh Hassan, Dja’de, Mureybet and to a lesser extent Halula) yielded δ^18^O_(v-smow)_ values much lower than locally expected values, measuring c. 12-15‰. Such low δ^18^O values would be consistent with individuals growing up in very high altitudes, a region much further inland and/or a region very far to the north of our study area. However, almost wholesale residential mobility of the communities at Cheikh Hassan, Dja’de and Mureybet from a different region is unlikely considering that the Sr ratios are consistent with local values. Moreover, the archaeological record gives no indications of such significant population mobility.

Other interpretations may involve changes in climate. An increase in δ^18^O values observed from the PPNB to Imperial Roman phases of neighboring Nevalı Çori has been interpreted as a warming trend on a larger temporal scale, in agreement with the paleoenvironmental records of the region [39]. Converted to phosphate δ^18^O_(v-smow)_, those values (n=28) range from 14.47 to 20.35‰, with the PPNB I-III and PN individuals falling at the lower end (δ^18^O 14.47-19.01‰). Although the authors observed no statistically significant differences among the PPNB sub-phases, the lowest measured values belonged to the PPNB I phase (δ^18^O 14.47-16.95‰), dated to ca. 8700-8300 BC, which is roughly contemporaneous with the studied Dja’de individuals. An equivalent gradual increase in δ^18^O values is not observed in our data (Fig. 3), which shows instead significant differences between the older (PPNA, Early and Middle PPNB) and more recent sites (Middle-Late PPNB, PN). An abrupt environmental change over the course of the Middle PPNB, between 8200 and 7550 BCE (after Mureybet and before Halula) does not explain the presence of one individual from Cheikh Hassan and one from Dja’de with higher δ^18^O values, closer to Halula and Sabi Abyad and within the modern δ^18^O baseline, and two additional individuals from Halula with low δ^18^O values, clustering with most of the values from Mureybet and Dja’de.

Another environmental input that may have altered δ^18^O_mw_ values is the Euphrates hydrological system. While modern irrigation and damming have caused an elevation of the δ^18^O values [35], other potential factors include access to meltwater run off from Quaternary glaciers or snow, resulting in lower δ^18^O_mw_ values. Lower-than-expected δ^18^O_mw_ values have been reported for the Early and Middle Bronze Ages, where more snowmelt from the Armenian Highlands and the Taurus Mountains feeding the Euphrates at that time has been argued [40]. We suspect a similar scenario was also the case in the Neolithic. In snowpack and glacier formation, the heavier isotope is concentrated in the solid phase (snow/ice) resulting in melting snow and ice (meltwaters) being ^18^O-depleted. Meltwaters have lower values in spring during the earliest phases of the snowmelt and higher values in summer and autumn, but the amount of meltwater released is much higher in the late summer and early autumn [41]. Therefore,^18^O-depleted waters could have been available at certain times of the year in some locales, perhaps even temporarily, leading to lower δ^18^O_mw_ values. In northern Syria during the last glacial maximum the snowline was c. 2800 m above sea level [42] but mountainous regions in northern Syria (e.g. Barsa, Hass, Kurd and Simeon mountains) are well below this height resulting in no permanent snowpack or glaciers. Even if snowpack did occur it would have been short lived and meltwater only available during spring. An alternative and more likely hypothesis is that meltwater introduced into the headwaters of the Euphrates in Türkiye from mountainous regions through much of the year made a major contribution to those waters. Meltwater substantially depleted in ^18^O can contribute up to 100% of river water in some months of the year [43]. This may point to one of two scenarios: (1) the inhabitants were able to access nearby meltwater sources with substantially lower δ^18^O values and tooth enamel values represent smoothed annual averages, or (2) for certain parts of the year (likely spring - autumn), meltwater contributed to the Euphrates, but inhabitants also drank from water sources (such as groundwater) without this contribution. This second scenario would suggest some individuals visiting and using different water sources during activities that formed part of the taskscape. Most of the individuals with low δ^18^O values also have Sr ratios consistent with local residence suggesting any mobility was within the same geological region, which is extensive, as other members of their community.

# **SUPPLEMENTARY FIGURES**


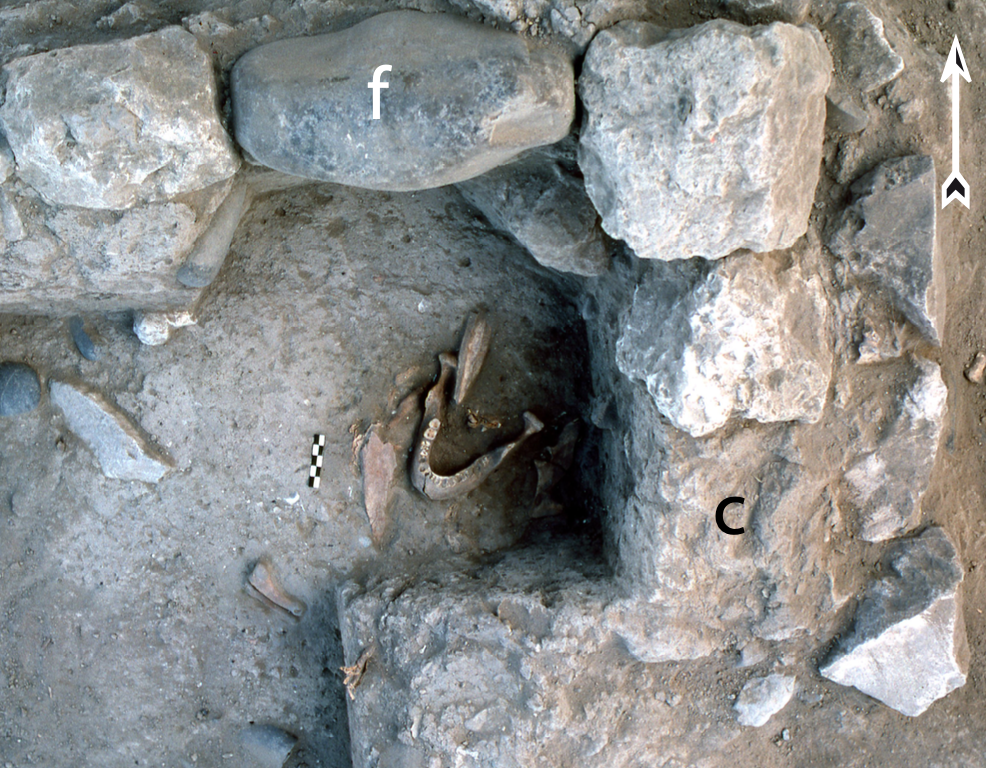

Supplementary Figure S1a: Dja’de *108-md a* *isolated*. Photo credit: French Mission of Dja'de el-Mughara.
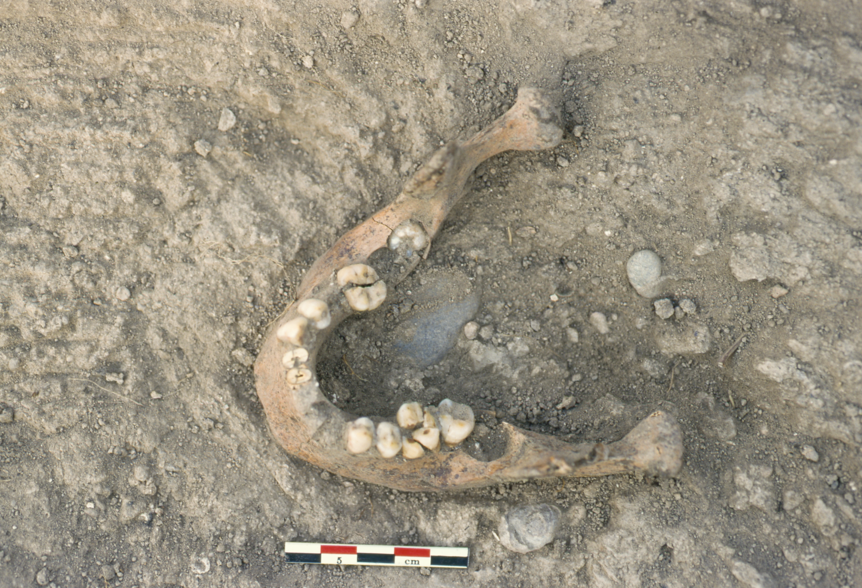

Supplementary Figure S1b: Dja’de 108-md c isolated. Photo credit: French Mission of Dja'de el-Mughara.


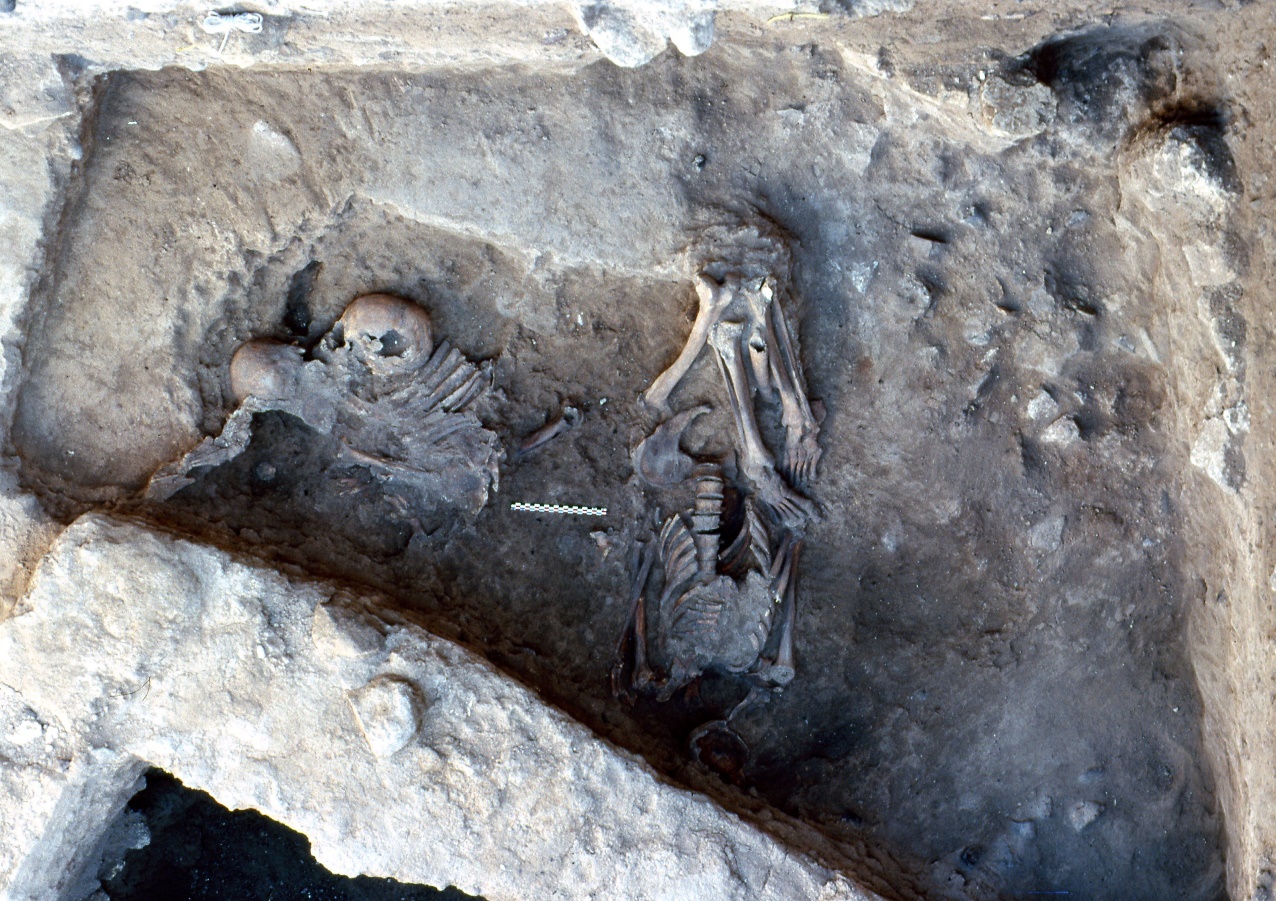

Supplementary Figure S2: Dja’de Dépôt 283 showing *283-sq C* supine in the center of the frame. Photo credit: French Mission of Dja'de el-Mughara.


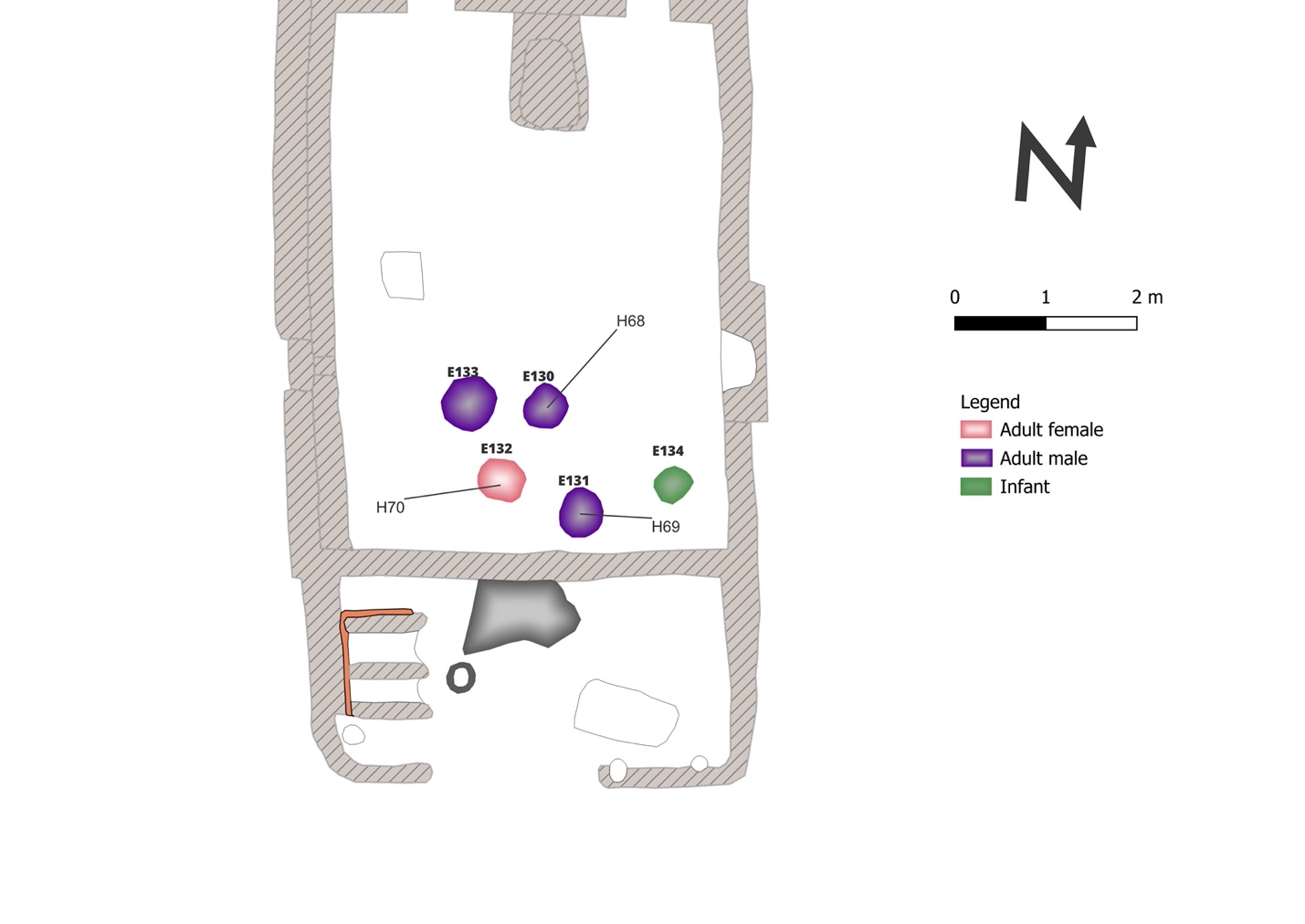

Supplementary Figure S3: Building DD at Halula and location of non-local individuals (H68a and H70). Image credit: GRAMPO-SAPPO/UAB/coord.M. Molist.


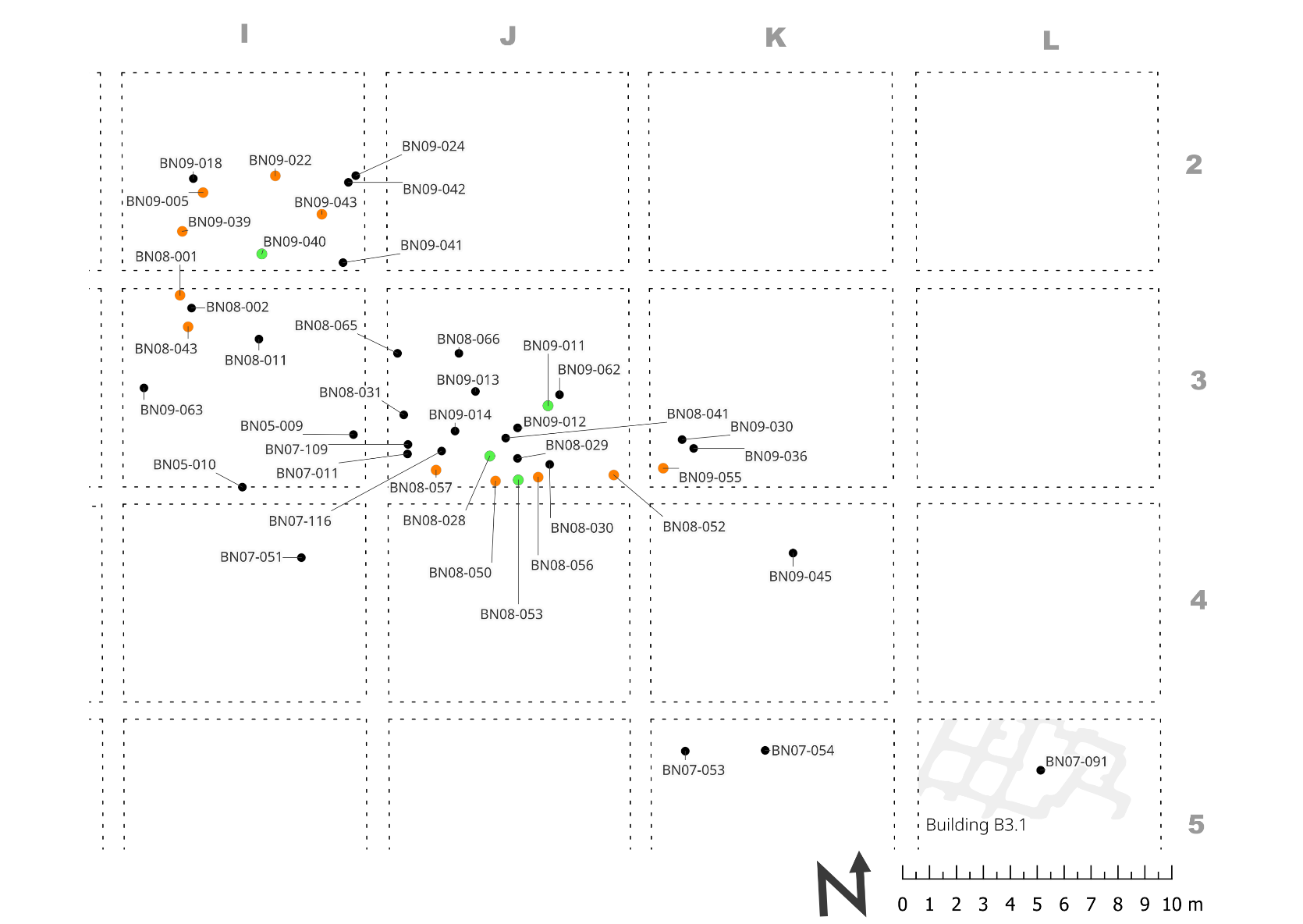


Supplementary Figure S4: Tell Sabi Abyad I, Operation III, level B3-C8. Orange: Locals, Green: non-locals. Black: not measured. Image credit: Sabi Abyad Team.

# **REFERENCES**

1. Chamel, B. Bioanthropologie s funéraires des populations néolithiques du Proche-Orient : L’impact de la néolithisation (Étude de sept sites syriens - 9820-et practique6000 cal. BC). (Université Lumière Lyon II, 2014).

2. Helmer, D. La domestication des animaux d’embouche dans le Levant Nord (Syrie du Nord et Sinjar) du milieu du IXe millenaire BP à la fin du VIIIe millenaire BP. Nouvelles données d’après les fouilles récentes. *Anthropozoologica* **20**, 41–54 (1994).

3. Abbès, F. Nouvelles recherches sur l’Obsidienne de Cheikh Hassan (Vallée de l’Euphrate, Syrie) au Néolithique : PPNA et PPNB ancien. *Syria* **78**, 5–17 (2001).

4. Coqueugniot, E. Dja’de (Syrie), Un village à la veille de la domestication (seconde moitié du IXe millénaire av. J.-C.). in *Premiers paysans du monde: naissances des agricultures [séminaire du Collège de France]* (Éditions Errance, Paris, 2000).

5. Willcox, G. Pre-Domestic Cultivation during the Late Pleistocene and Early Holocene in the Northern Levant. in *Biodiversity in Agriculture* (eds. Gepts, P. et al.) 92–109 (Cambridge University Press, 2012). doi:10.1017/CBO9781139019514.007.

6. Willcox, G., Fornite, S. & Herveux, L. Early Holocene cultivation before domestication in northern Syria. *Veget Hist Archaeobot* **17**, 313–325 (2008).

7. Gourichon, L. Faune et saisonnalité: L’organisation temporelle des activités de subsistance dans l’Epipaléolithique et le Néolithique précéramique du Levant nord (Syrie). (Université Lumière Lyon II, 2004).

8. Chamel, B. & Coqueugniot, E. Human Self-perception and Self-expression during the 9th Millennium calBC: Funerary Practices and Symbolic Meaning of the Human Representations at Dja’de el-Mughara (Syria). in *Human Iconography and Symbolic Meaning in Near Eastern Prehistory: Proceedings of the Workshop held at 10th ICAANE in Vienna, April 2016* (eds. Becker, J., Beuger, C. & Müller-Neuhof, B.) (Verlag der österreichischen Akademie der Wissenschaften, 2019). doi:10.2307/j.ctvvh865z.

9. Stordeur, D., Brenet, M., Der Aprahamian, G. & Roux, J. C. Les bâtiments communautaires de Jerf el Ahmar et Mureybet Horizon PPNA (Syrie). *paleo* **26**, 29–44 (2000).

10. Cauvin, J. The excavations of Mureybet (1971-1974) and their significance for the origins of sedentarization in the Near East. *Annual American School of Oriental Research* **44**, 19–48 (1977).

11. Gourichon, L. & Helmer, D. Étude archéozoologique de Mureybet. in *Le site neolithique de Tell Mureybet (Syrie Du Nord), En hommage à Jacques Cauvin* (ed. Ibáñez, J. J.) 115–228 (BAR International Series, Oxford - Lyon, 2008).

12. Molist, M. Tell Halula (Aleppo). in *A history of Syria in one hundred sites* (eds. Tsuneki, A. & Kanjou, Y.) 54–56 (Archaeopress, Oxford, 2016).

13. Molist, M. *et al.* Tell Halula (Euphrates Valley, Syria): New data from the Late Neolithic settlement. in *Interpreting the late Neolithic of Upper Mesopotamia* (eds. Nieuwenhuyse, O., Bernbeck, R., Akkermans, P. & Rogash, J.) (Brepols, Turnhout, 2013).

14. Araus, J. L., Ferrio, J. P., Voltas, J., Aguilera, M. & Buxó, R. Agronomic conditions and crop evolution in ancient Near East agriculture. *Nat Commun* **5**, 3953 (2014).

15. Saña, M. & Tornero, C. Consumption of animal resources at the sites of Akarçay Tepe and Tell Halula (Middle Euphrates Valley, 8th-6th millennia cal. BC). in *Archaeozoology of the Near East VIII. Actes des huitièmes Rencontres Internationales d’Archéozoologie de l’Asie du Sud-Ouest et des régions adjacentes* 153–167 (Travaux de la Maison de l’Orient et de la Méditerranée, Lyon: Maison de l’Orient et de la Méditerranée Jean Pouilloux, 2008).

16. Tornero, C., Molist, M. & Saña, M. Adoption, Intensification and Manipulation of Sheep Husbandry at Tell Halula, Syria during the Middle to Late PPNB. in *Bones and Identity Zooarchaeological Approaches to Reconstructing Social and Cultural Landscapes in Southwest Asia* (eds. Marom, N., Yeshurun, L., Weissbrod, L. & Bar-Oz, G.) 73–86 (Oxbow Books, 2016).

17. Molist, M. *et al.* New Metallurgic Findings from the Pre-Pottery Neolithic: Tell Halula (Euphrates Valley, Syria). *paleo* **35**, 33–48 (2009).

18. Ortiz, A., Chambon, P. & Molist, M. “Funerary bundles” in the PPNB at the archaeological site of Tell Halula (middle Euphrates valley, Syria): analysis of the taphonomic dynamics of seated bodies. *Journal of Archaeological Science* **40**, 4150–4161 (2013).

19. Guerrero, E., Molist, M., Kuijt, I. & Anfruns, J. Seated Memory: New Insights into Near Eastern Neolithic Mortuary Variability from Tell Halula, Syria. *Current Anthropology* **50**, 379–391 (2009).

20. Akkermans, P. M. M. G. Living Space, Temporality and Community Segmentation: Interpreting Late Neolithic Settlement in Northern Syria. in *Interpreting the late Neolithic of Upper Mesopotamia* (eds. Nieuwenhuyse, O. P., Bernbeck, R., Akkermans, P. M. M. G. & Rogash, J.) 63–75 (Brepols, Turnhout, 2013).

21. Akkermans, P. M. M. G. *et al.* Investigating the Early Pottery Neolithic of Northern Syria: New Evidence from Tell Sabi Abyad. *American Journal of Archaeology* **110**, 123–156 (2006).

22. Nieuwenhuyse, O. P., Akkermans, P. M. M. G. & Plicht, J. van der. Not so coarse, nor always plain – the earliest pottery of Syria. *Antiquity* **84**, 71–85 (2010).

23. Van Der Plicht, J., Akkermans, P. M. M. G., Nieuwenhuyse, O., Kaneda, A. & Russell, A. Tell Sabi Abyad, Syria: Radiocarbon Chronology, Cultural Change, and the 8.2 ka Event. *Radiocarbon* **53**, 229–243 (2011).

24. Cavallo, C. *Animals in the Steppe: A Zooarchaeological Analysis of Later Neolithic Tell Sabi Abyad, Syria: A Zooarchaeological Analysis of Later Neolithic Tell Sabi Abyad, Syria*. (University of Michigan Press, Ann Arbor, MI, 2000). doi:10.30861/9781841711539.

25. Russell, A. Retracing the Steppes: A Zooarchaeological Analysis of Changing Subsistence Patterns in the Late Neolithic at Tell Sabi Abyad, Northern Syria, c. 6900 to 5900 BC. (Leiden University, Leiden, 2010).

26. Akkermans, P. M. M. G. & Duistermaat, K. Of storage and nomads. The sealings from Late Neolithic, Sabi Abyad, Syria. *paleo* **22**, 17–44 (1996).

27. Verhoeven, M. *An Archaeological Ethnography of a Neolithic Community: Space, Place and Social Relations in the Burnt Village at Tell Sabi Abyad, Syria*. (Nederlands Historisch-Archaeologisch Instituut te Instanbul [sic] ; Nederlands Instituut voor het Nabije Oosten [distributor], [Istanbul] : Leiden, Nederland, 1999).

28. *Relentlessly Plain: Seventh Millennium Ceramics at Tell Sabi Abyad, Syria*. (Oxbow Books, 2018). doi:10.2307/j.ctvh1dhb9.

29. Nieuwenhuyse, O. P. Feasting in the Steppe - Late Neolithic Ceramic Change and the Rise of the Halaf. in *Proceedings of the 5th International Congress on the Archaeology of the Ancient Near East* (eds. Córdoba, J., Molist, M., Peréz, C., Rubio, I. & Martínez, S.) (Centro Superior de Estudios sobre el Oriente Próximo y Egipto, Madrid, 2008).

30. Nieuwenhuyse, O. P. & Akkermans, P. M. M. G. Transforming the Upper Mesopotamian Landscape in the Late Neolithic. in *Concluding the Neolithic* (ed. Marciniak, A.) 101–136 (Lockwood Press, 2019). doi:10.5913/87913.CN.05.

31. Plug, J.-H., Van Der Plicht, J. & Akkermans, P. M. M. G. Tell Sabi Abyad, Syria: Dating of Neolithic Cemeteries. *Radiocarbon* **56**, 543–554 (2014).

32. Plug, J.-H. Uncovering a Community - Lifestyles and Death Ways at Late Neolithic Tell Sabi Abyad, Syria. (University of Liverpool, Liverpool, 2021).

33. Akkermans, P. M. M. G. Burying the Dead in Late Neolithic Syria. in *Proceedings of the 5th International Congress on the Archaeology of the Ancient Near East* (eds. Córdoba, J. et al.) (Centro Superior de Estudios sobre el Oriente Próximo y Egipto, Madrid, 2008).

34. Kattan, Z. Factors controlling stable isotopes variability in precipitation in Syria: Statistical analysis approach. *J Earth Syst Sci* **128**, 151 (2019).

35. Kattan, Z. Estimation of evaporation and irrigation return flow in arid zones using stable isotope ratios and chloride mass-balance analysis: Case of the Euphrates River, Syria. *Journal of Arid Environments* **72**, 730–747 (2008).

36. Pollard, A. m., Pellegrini, M. & Lee-Thorp, J. a. Technical note: Some observations on the conversion of dental enamel δ18op values to δ18ow to determine human mobility. *American Journal of Physical Anthropology* **145**, 499–504 (2011).

37. Gat, J. R. Comments on the Stable Isotope Method in Regional Groundwater Investigations. *Water Resources Research* **7**, 980–993 (1971).

38. Al-Charideh, A. Environmental isotope study of groundwater discharge from the large karst springs in West Syria: a case study of Figeh and Al-sin springs. *Environ Earth Sci* **63**, 1–10 (2011).

39. Wang, X. *et al.* Isotopic and DNA analyses reveal multiscale PPNB mobility and migration across Southeastern Anatolia and the Southern Levant. *Proceedings of the National Academy of Sciences* **120**, e2210611120 (2023).

40. Tomczyk, J., Wierzbowski, H. & Zalewska, M. Stable Isotope Record of Human and Sheep Enamel Carbonate from the Ancient Middle Euphrates Valley (Syria). *International Journal of Osteoarchaeology* **26**, 599–609 (2016).

41. McGuire, K. & McDonnell, J. Stable Isotope Tracers in Watershed Hydrology. in *Stable Isotopes in Ecology and Environmental Science* (eds. Michener, R. & Lathja, K.) 334–374 (Blackwell Publishing, London, 2007). doi:10.1002/9780470691854.ch11.

42. Sarıkaya, M. A., Çiner, A. & Zreda, M. Chapter 30 - Quaternary Glaciations of Turkey. in *Developments in Quaternary Sciences* (eds. Ehlers, J., Gibbard, P. L. & Hughes, P. D.) vol. 15 393–403 (Elsevier, 2011).

43. Rets, E. *et al.* How and when glacial runoff is important: Tracing dynamics of meltwater and rainfall contribution to river runoff from headwaters to lowland in the Caucasus Mountains. *Science of The Total Environment* **927**, 172201 (2024).

44. Henderson, J., Evans, J. & Barkoudah, Y. The roots of provenance: glass, plants and isotopes in the Islamic Middle East. *Antiquity* **83**, 414–429 (2009).

45. Santana, J. *et al.* Multi-isotope evidence of population aggregation in the Natufian and scant migration during the early Neolithic of the Southern Levant. *Sci Rep* **11**, 11857 (2021).

46. Alt, K. W. *et al.* Earliest Evidence for Social Endogamy in the 9,000-Year-Old-Population of Basta, Jordan. *PLoS ONE* **8**, e65649 (2013).

47. Knipper, C., Gresky, Julia, & Benz, Marion. Local People or Masked Mobility: Results of Strontium Isotope Analysis of Human Teeth. in *Death in Ba’ja: Sepulchral Identity and Symbolism in an Early Neolithic Community of the Transjordanian Highlands. Household and Death in Ba’ja 2* (eds. Benz, M., Gresky, J., Purschwitz, C. & Gebel, H. G. K.) (ex oriente, Berlin, 2023). doi:10.11588/propylaeum.1224.

48. Wang, X. *et al.* Isotopic and proteomic evidence for communal stability at Pre-Pottery Neolithic Jericho in the Southern Levant. *Sci Rep* **13**, 16360 (2023).

49. Pearson, J. *et al.* Mobility and kinship in the world’s first village societies. *Proceedings of the National Academy of Sciences* **120**, e2209480119 (2023).
